# The ACBD3 protein coordinates ER-Golgi contacts to enable productive TBEV infection

**DOI:** 10.1128/jvi.02224-24

**Published:** 2025-04-10

**Authors:** Wai-Lok Yau, Marie B. A. Peters, Sebastian Rönfeldt, Marie N. Sorin, Richard Lindqvist, Lauri I. A. Pulkkinen, Lars-Anders Carlson, Anna K. Överby, Richard Lundmark

**Affiliations:** 1 Department of Medical and Translational Biology, SciLifeLab, Umeå University 8075, Umeå, Sweden; 2 The Laboratory for Molecular Infection Medicine Sweden (MIMS), Umeå University 8075, Umeå, Sweden; 3 Department of Clinical Microbiology, Umeå University 8075, Umeå, Sweden; 4 Department of Medical Biochemistry and Biophysics, Umeå University 8075, Umeå, Sweden; University of North Carolina at Chapel Hill, Chapel Hill, North Carolina, USA

**Keywords:** *Orthoflavivirus*, flavivirus, NS4B, ACBD3, host-pathogen interaction, replication organelles, ER exit sites, ERES-Golgi contact

## Abstract

Flavivirus infection involves extensive remodeling of the endoplasmic reticulum (ER), which is key to both the replication of the viral RNA genome as well as the assembly and release of new virions. However, little is known about how viral proteins and host factors cooperatively facilitate such a vast transformation of the ER, and how this influences the different steps of the viral life cycle. In this study, we screened for host proteins that were enriched in close proximity to the tick-borne encephalitis virus (TBEV) protein NS4B and found that the top candidates were coupled to trafficking between ER exit sites (ERES) and the Golgi. We characterized the role of ACBD3, one of the identified proteins, and showed that it promotes TBEV infection. Depletion of ACBD3 inhibited virus replication and resulted in abnormal transformation of the ER, leading to reduced virion release. ACBD3’s proviral mechanism did not involve the recruitment of PI4PK as previously described for enteroviruses. Instead, productive TBEV infection required the full-length ACBD3, which localizes to ER-Golgi contact sites together with NS4B. We propose that NS4B and ACBD3 promote replication by coordinating the transformation of the ER, which is required for RNA replication and particle release. The transformation involves direct coupling to the Golgi which facilitates efficient virion transport.

**IMPORTANCE:**

Flaviviruses like tick-borne encephalitis have significant effects on human health. During flavivirus infection, the viral particles enter the host cells and transform the endoplasmic reticulum (ER), which is a membranous organelle and the main site of cellular protein synthesis. Although this is critical for successful infection, the details of the process are unknown. Here, we found that the viral protein NS4B and the host protein ACBD facilitate this transformation by ensuring that the ER is coupled to the Golgi apparatus, the organelle responsible for transporting material out of the cell. TBEV uses ACBD3 to guarantee that the connection sites between the transformed ER and the Golgi remain functional so that RNA is replicated and the produced viral particles are exported from the cell and can infect further cells. Our work sheds light both on the basic biology of flavivirus infection, and virus-induced remodeling of membranous organelles.

## INTRODUCTION

Flaviviruses (genus *Orthoflavivirus*) are enveloped positive-sense single-stranded RNA viruses (1). This family includes medically relevant members such as Japanese encephalitis virus (JEV), West Nile virus (WNV), and tick-borne encephalitis virus (TBEV) (2). Flavivirus virions enter the host cell via endocytosis, which is followed by the fusion of the viral envelope with the endosomal membrane. This releases the genome that is directly translated as single polyprotein at the endoplasmic reticulum (ER). The polyprotein is post-translationally processed by host and viral proteases into three structural proteins, capsid (C), pre-membrane (prM), and envelope (E), as well as seven non-structural (NS) proteins (NS1, NS2A, NS2B, NS3, NS4A, NS4B, and NS5) (3). The expression of the viral proteins allows the virus to hijack the cell and repurpose the ER and the secretory pathway for the viral genome replication, virion assembly, and export (3–5). The generation of an extensively transformed ER membrane (TERM) is a hallmark of flavivirus infection and includes small ER membrane invaginations and replication organelles (ROs), where the new genome copies are synthesized (6–13). The newly synthesized RNA genomes exit the ROs and are either translated to yield more viral proteins or packaged into virions. The genomes that end up packaged interact with the C protein on the ER membrane to form nucleocapsids (NCs) that then bud into the lumen of the ER and acquire the lipid envelope and the embedded prM and E proteins (6, 7, 14, 15). This process yields immature virions that need to undergo protease cleavage and pH-mediated conformational changes to produce infectious virions that are then secreted from the cell (16–20).

Although TERM formation and the remodeling of the secretory pathway are crucial for productive flavivirus infection, the details of the process are poorly understood. The TERM needs to support viral RNA synthesis and coordinate the export of virions while retaining unassembled structural proteins and the components of the viral RNA replication machinery. In healthy cells, anterograde and retrograde trafficking between ER and Golgi is concentrated to ER exit sites (ERES) where the coat protein complex II (COPII) machinery facilitates ER-Golgi trafficking and COPI vesicles mediate transport from Golgi to the ER (21, 22). These processes are intimately coupled and dependent upon each other and multiple proteins are involved in regulating this intricate balance of membrane trafficking. Interestingly, recent data show that ERES-Golgi contacts can be modified to facilitate the trafficking of large proteins (23–27). Modification of these contacts has also been described during bacterial infection (28). Therefore, it is likely that these sites are also exploited by the flaviviral NS proteins by specifically interacting with the proteins that regulate ERES-Golgi contact sites (12, 29–31). One of the proteins responsible for this may be NS4B. It is a small integral membrane protein that interacts with viral and host proteins to take part in acritical role in the transformation of the ER as well as RO generation (29, 31, 32). NS4B is detected throughout the entire TERM, and its precise contribution to membrane remodeling as well as the plethora of host proteins it interacts with has not been thoroughly investigated.

In this study we focused on the medically relevant TBEV and utilized the model virus LGTV (Langat virus, low-pathogenic TBEV-like virus) to investigate the factors involved in the generation of TERM during infection (1). We used ascorbate peroxidase (APEX2)-based NS4B screening and identified several ERES proteins in close proximity to NS4B, including the *cis*-Golgi resident acyl-coenzyme A binding domain containing protein 3 (ACBD3). This suggested that NS4B has additional functions in the TERM outside of ROs and their generation. We characterized the role of ACBD3 and show that it is a proviral host factor for both TBEV and LGTV. ACBD3 depletion reduced bulk viral RNA synthesis and virion release and resulted in altered TERM morphology with excessive accumulation of viral proteins at the TERM. Detailed characterization showed that ACBD3 is specifically needed both for RNA replication and particle egress. Our results suggest that ACBD3 is exploited by flaviviruses to coordinate TERM generation to facilitate successful infection from RNA synthesis to virion assembly.

## RESULTS

### Screen for NS4B-proximal host factors identified multiple ERES proteins

**Fig 1:**
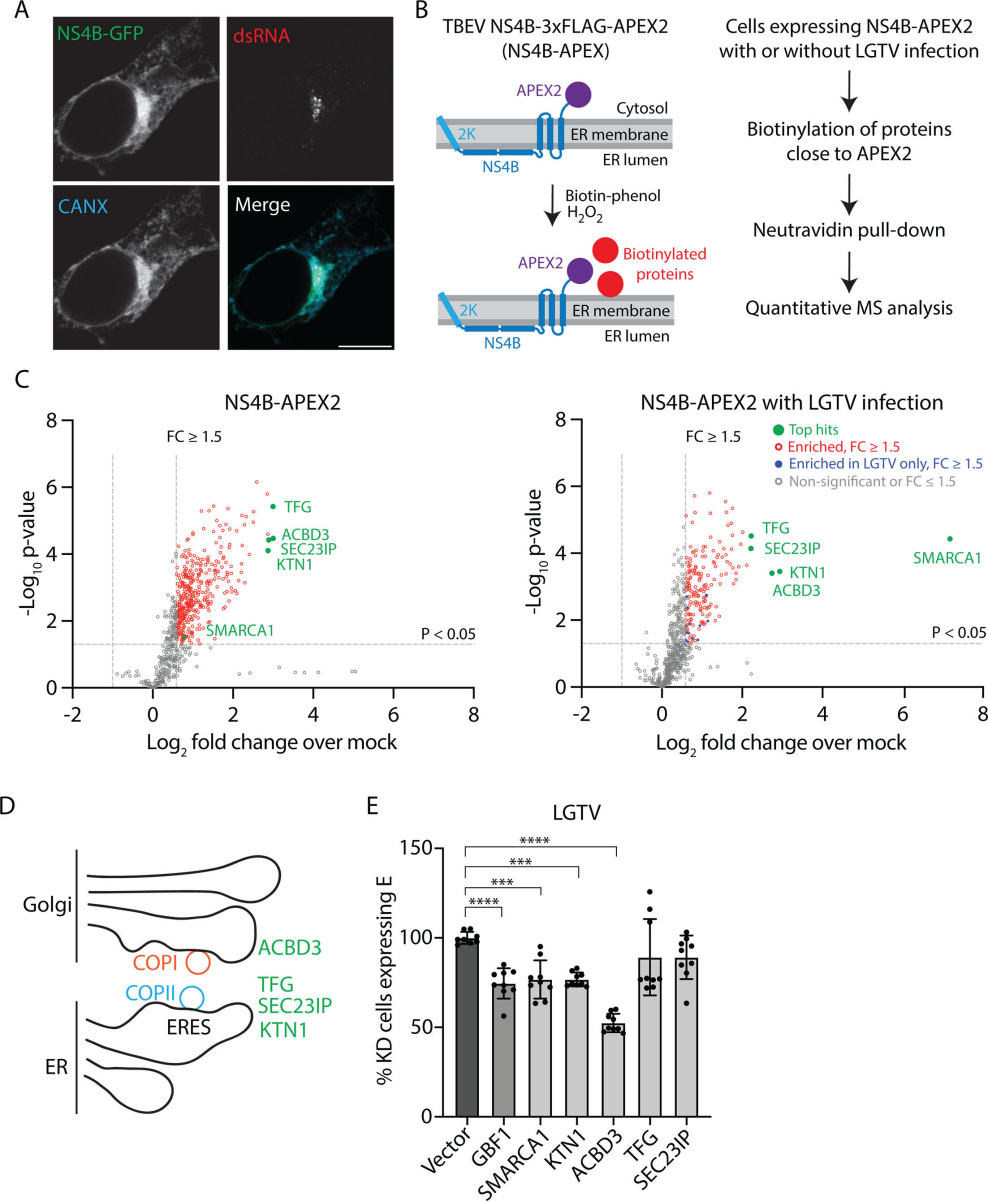
Identification of ACBD3 as a host factor in NS4B-APEX2 proximal protein analysis. (**A**) Confocal fluorescence micrographs of HEK293T cells transiently expressing TBEV NS4B-GFP infected with LGTV (MOI 10, 16 h.p.i.) and stained with anti-calnexin (CANX, ER marker) antibodies and anti-dsRNA antibodies. Scale bar, 10 µm. (**B**) Schematic illustration of the NS4B-APEX2 proximal protein biotinylation screen used in this study. (**C**) Volcano plots of the proteins identified in the NS4B-APEX2 proximal protein biotinylation screen in NS4B-APEX2 cells (left) and NS4B-APEX2 cells infected with LGTV (MOI 10, 16 h.p.i.) (right). Proteins are indicated by dots color-coded based on the FC and *P*-values. Three biological replicates per condition were analyzed, and the *P*-value was calculated using unpaired *t*-test. (**D**) Schematic illustration of ERES-Golgi contacts, and the top hit proteins ACBD3, TFG, SEC23IP, and KTN1 (**E**) Quantification of the percentage of GFP-positive KD HEK293T cells expressing E protein at 24 h after LGTV infection (MOI 1). The KD proteins are indicated. Mean ± SD of 3 independent experiments. One-way ANOVA with Dunnett multiple tests, ***P < 0.005, ****P < 0.0001.

To identify host factors that are involved in transforming the ER membrane during infection, we used APEX2-based biotinylation to screen for proteins that localize close to TBEV NS4B (33). First, we confirmed that recombinantly expressed NS4B with the 2K sorting signal localizes in the ER of uninfected HEK293T cells (Fig. S1A) and across the entire TERM in transfected cells infected with LGTV (Fig. 1A). We generated a Flp-In T-REx HEK293T cell line with inducible expression of TBEV NS4B with an APEX2 tag in the cytosolic C terminus (NS4B-APEX2). Next, we induced the expression of APEX2-tagged NS4B and supplemented the cells with biotin-phenol (BP) and hydrogen peroxide (H_2_O_2_), which causes the biotinylation of proteins in the vicinity of the APEX2 tag (33). As protein abundance and localization can change as a result of virus infection, we also infected NS4B-APEX2 cells with LGTV at multiplicity of infection (MOI) of 10 for 2 h before inducing NS4B expression and treating the cells as above. The biotinylated proteins were purified with neutravidin beads and identified using quantitative liquid chromatography-mass spectrometry (LC-MS) analysis (Fig. 1B; Fig. S1B and C).

We used a significance cutoff of *P*-value < 0.05 and a fold-change of abundance (FC) cutoff of 1.5 to identify the "hits" of the screen. We found 381 hit proteins in the non-infected cells and 190 in the infected cells, with 173 proteins identified in both experiments (Fig. 1C; Table S1). Interestingly, our highest-scoring hits included the ERES proteins ACBD3, TFG, SEC23IP, and KTN1 (Fig. 1C and D) (34–40). Additionally, 17 other proteins identified in the screen are also involved in ER-Golgi trafficking according to Search Tool for the Retrieval of Interacting Genes/Proteins (STRING) functional enrichment analysis (Table S1). Furthermore, the transcription factor SMARCA1, which is usually a nuclear protein (41), was greatly enriched in infected cells compared with the uninfected cells, suggesting that its localization changes during infection (Fig. 1C).

We moved forward with ACBD3, TFG, SEC23IP, KTN1, and SMARCA1. To test if these proteins are important for LGTV infection, we used GFP-reporter assisted CRISPR-Cas9 gene targeting to reduce the expression of the proteins. In this screen, we did not select for knocked out (KO) cell clones, and therefore, we refer to these cell populations as knocked down (KD). We quantified the fraction of successfully transfected cells via flow cytometry and confirmed the reduction in protein levels by immunoblotting (Fig. S1D and E). KD cell populations were infected with LGTV at an MOI of 1 and stained with anti-E antibody at 24 h post-infection (h.p.i). The fraction of successfully transfected (GFP-positive) cells that were infected (E-expressing) was quantified by flow cytometry. We used GBF1 as a positive control since it is a known host factor for LGTV (42). GBF1, SMARCA1, KTN1, and ACBD3 KD significantly reduced the fraction of infected cells compared with vector-only control, whereas the effects of TFG and SEC23IP KD were not statistically significant (Fig. 1E). In the case of TFG, this may be caused by the low KD efficiency (Fig. S1E).

### ACBD3 is located at the contact sites between the TERM and Golgi

**Fig 2:**
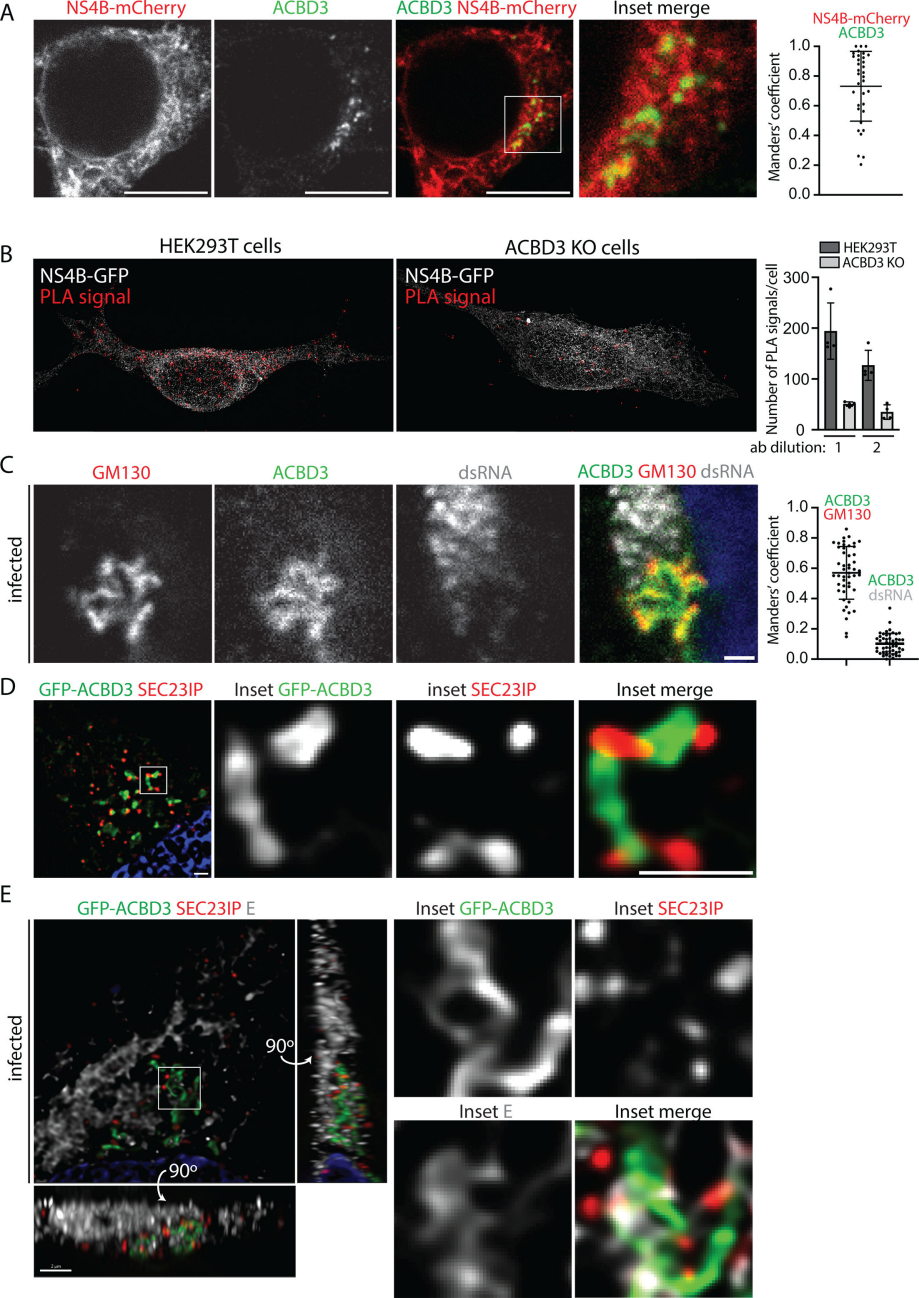
ACBD3 localization in healthy and infected cells. (**A**) Confocal fluorescence micrographs of HEK293T cells transiently expressing NS4B-mCherry and stained with anti-ACBD3 antibodies. Scale bars, 10 µm. Manders’ coefficient between the ACBD3 and NS4B signals is shown on the right-hand side as mean ± SD. (**B**) Fluorescent SIM images of HEK293T and ACBD3 KO cells transfected with NS4B-GFP and processed using the PLA providing a signal for the close proximity between antibodies against GFP and ACBD3 (ab dilution 1). Quantification of the number of PLA signal events per cell is shown on the right. Ab dilution 1: anti-GFP 1:200, anti-ACBD3 1:500; ab dilution 2: anti-GFP 1:100, anti-ACBD3 1:1000. (**C**) Confocal fluorescence micrographs (single Z frame) of LGTV-infected (MOI 1, 16 h.p.i.) HEK293T cells and stained with antibodies against ACBD3, GM130, and dsRNA. Scale bar, 1 µm. Manders’ coefficient between the indicated signals is shown on the right-hand side as mean ± SD. (**D**) SIM fluorescence micrographs (100 nm resolution) of HEK293T cells transiently expressing GFP-ACBD3 and stained with anti-SEC23IP antibodies. Scale bars, 1 µm. (**E**) SIM fluorescence micrographs (100 nm resolution) of HEK293T cells transiently expressing GFP-ACBD3 at 16 h after LGTV infection (MOI 1) stained with anti-SEC23IP and anti-E antibodies. Scale bars, 1 µm. The left-hand panels show the image stack projected along the Z, Y, and X axes. The right-hand panels show a single Z frame of the inset area.

As reduced ACBD3 expression had the greatest effect on LGTV infection, we focused on this protein. First, we used confocal microscopy to characterize the cellular localization of ACBD3 and NS4B. We transfected the HEK293T cells with a plasmid for transient NS4B-mCherry expression and stained the cells with an anti-ACBD3 antibody. ACBD3 was detected as punctae that colocalized with the NS4B signal in the ER (Fig. 2A). To verify that NS4B was located close to ACBD3, we used proximity ligation assay (PLA), in which a signal is generated when antibodies against two proteins are within a distance of 40 nm or less. As control, we used a stable ACBD3 knockout (KO) HEK293T cell line previously characterized and here referred to as ACBD3 KO cells (43). Comparing WT and ACBD3 KO cells, the PLA signal was significantly higher in WT cells, showing that NS4B is found proximal to ACBD3 (Fig. 2B), in agreement with being identified in the APEX2 screen. In agreement with the proposed localization of ACBD3 at the ERES-Golgi interface, we found that ACBD3 partially colocalized with GM130, a *cis*-Golgi marker, which in turn partially colocalized with SEC23IP (Fig. S2A and B). A similar overlap in localization between ACBD3, GM130, and SEC23IP was observed in cells infected with LGTV at an MOI of 1 for 16 h (Fig. 2C; Fig. S2C), suggesting that the ERES-Golgi contact sites likely remain intact even after the remodeling of the ER. Interestingly, ACBD3 did not co-localize with dsRNA, which marks the sites of active RNA replication, but it was detected next to these sites (Fig. 2C). Furthermore, NS3 and E were detected in a wide area within which ACBD3, SEC23IP, dsRNA, and GM130 localize (Fig. S2C through E).

To visualize the organization of the ERES-Golgi contact sites at a higher resolution, we used structured illumination microscopy (SIM). We transfected HEK293T cells with GFP-ACBD3 and stained the cells with a SEC23IP antibody. We also infected the transfected cells with LGTV for 16 h at an MOI of 1 and stained the cells for SEC23IP and E. In both infected and non-infected cells, the punctate SEC23IP signal was detected in close proximity to the GFP-ACBD3-positive membrane tubules (Fig. 2D and E; Movie S1). In infected cells, E protein was also detected at these sites, and three-dimensional analysis showed that the proteins form a complex interwoven network of elongated membrane structures (Fig. 2E; Movie S2). These data suggest that during infection, NS4B may act with ACBD3 to coordinate the remodeling of the cellular membranes by bringing together the TERM (positive for NS3 and E), ERES (positive for SEC23IP), and the *cis*-Golgi (positive for GM130).

### Loss of ACBD3 leads to reduced replication, virus particle release, and altered TERM morphology

**Fig 3:**
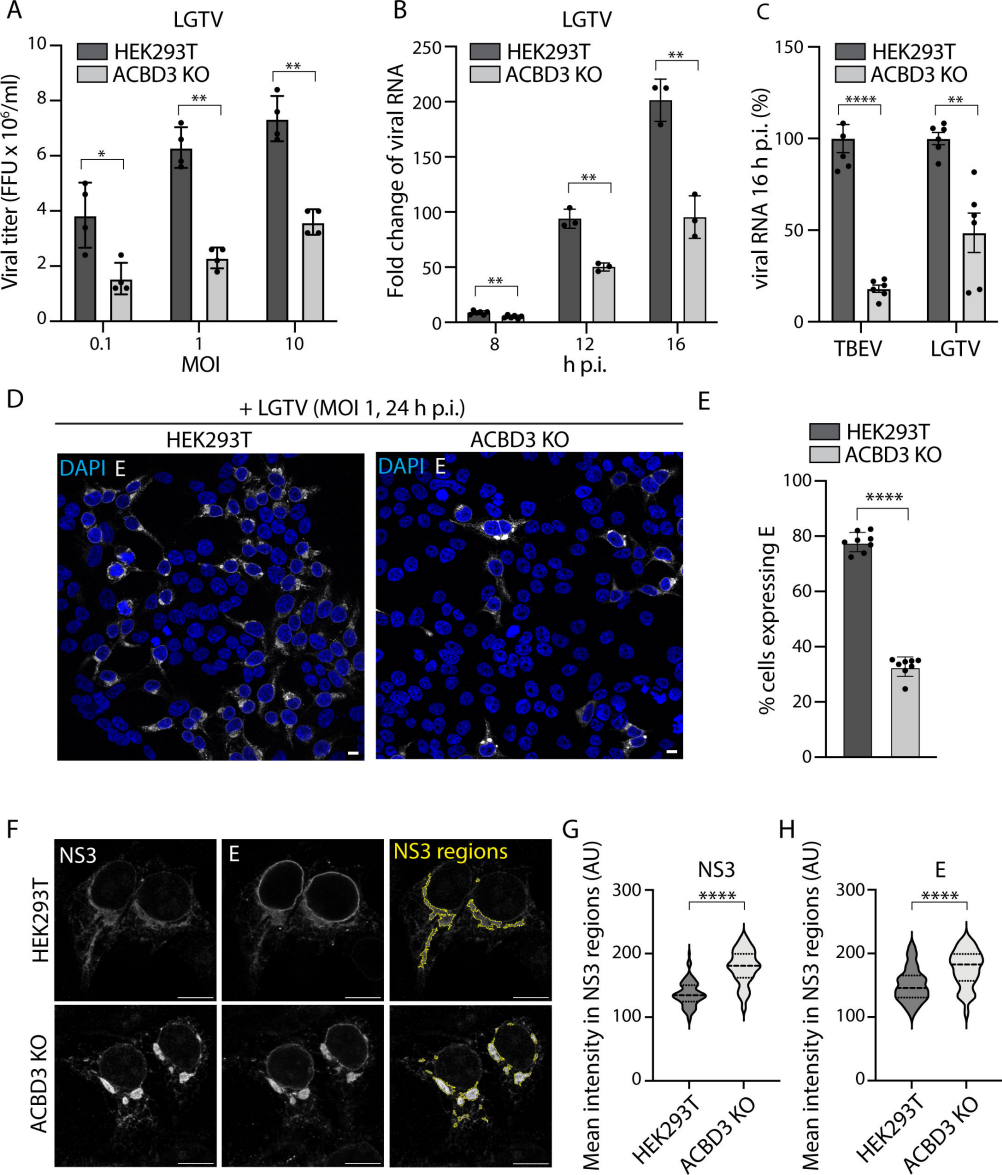
ACBD3 promotes flavivirus infection. (**A**) Quantification of the viral titers of the supernatant from LGTV-infected (24 h.p.i.) HEK293T cells and ACBD3 KO cells by focus-forming assay. Mean ± SD of 4 biological replicates. Unpaired *t*-test, (**B**) Viral RNA in LGTV-infected (MOI 1) HEK293T and ACBD3 KO cells quantified by RT-qPCR and normalized to actin and to the 2 h post-infection (input) using the ∆∆Ct method and given as fold change. Mean ± SD of 3 biological replicates. Unpaired *t*-test. (**C**) Quantification of relative amounts of TBEV and LGTV RNA in infected (MOI 1, 16 h.p.i.) HEK293T cells and ACBD3 KO cells compared to HEK293T cells by RT-qPCR and normalized to actin. Data were normalized to actin as in (**B**) and to HEK293T cells for comparison. Mean ± SD of 6 biological replicates. Unpaired *t*-test. (**D**) Confocal fluorescence micrographs of LGTV-infected (MOI 1, 24 h.p.i.) HEK293T and ACBD3 KO cells stained with anti-E antibodies and DAPI. Scale bar, 10 µm. (**E**) Quantification of the percentage of HEK293T and ACBD3 KO cells expressing E protein at 24 h after LGTV infection (MOI 1). Mean ± SD of at least seven biological replicates. Unpaired *t*-test. (**F**) Confocal fluorescence micrographs of LGTV-infected (MOI 1, 16 h.p.i.) HEK293T and ACBD3 KO cells stained with anti-NS3 antibodies and anti-E antibodies. Yellow lines in the right-hand panels indicate the identified NS3 regions (see Materials and Methods). Scale bar, 10 µm. (**G–H**) Quantification of the mean fluorescence intensities of NS3 (**G**) and E (**H**) within the identified NS3 regions. Data are presented in violin plots with median and quartiles indicated by dashed and dotted lines, respectively. Number of NS3 regions analyzed, HEK293T (*n* = 95), ACBD3 KO (*n* = 103). Unpaired *t*-test. *P < 0.05, **P < 0.005, ****P < 0.0001 in (**A–C**), (**E**), and (**G–H**).

As the correct TERM formation is critical for both virion production and RNA replication, we investigated how ACBD3 depletion affects these processes using ACBD3 knockout (KO) HEK293T cells (43). We confirmed that in ACBD3 KO cells, the production of infectious LGTV particles was reduced by quantification of the focus-forming assays (MOI: 0.1, 1, 10; 24 h.p.i.) (Fig. 3A). Additionally, immunoblot analysis showed that the expression of LGTV NS3, E, and prM was reduced in infected ACBD3 KO cells (Fig. S3A). Furthermore, we used quantitative reverse-transcription PCR (RT-qPCR) to show that ACBD3 KO cells produced significantly less LGTV RNA than WT cells at 8, 12, and 16 h after infection at MOI 1 (Fig. 3B). We also used RT-qPCR analysis to quantify the effects of ACBD3 KO on other flavivirus species. We infected ACBD3 KO and WT HEK293T cells with LGTV, TBEV, WNV, and JEV and quantified the relative effects of ACBD3 KO at 16 h.p.i. and an MOI of 1. Interestingly, ACBD3 KO had an even bigger effect on TBEV compared with LGTV (Fig. 3C). However, the effect on WNV was modest, and JEV was not affected at all (Fig. S3B). This suggests that the role of ACBD3 in mosquito-borne flaviviruses is minor, and we continued to focus on tick-borne species.

Although ACBD3 KO had multiple effects on viral infection, it also reduced the number of infected cells quantified by fluorescence microscopy. After both LGTV and TBEV infections (24 h.p.i., MOI 1), a smaller fraction of ACBD3 KO cells was positive for E compared with WT HEK293T cells (Fig. 3D and E; Fig. S3C). The fraction of E-positive ACBD3 KO cells was confirmed to lack ACBD3 using fluorescence microscopy (Fig. S3D). Interestingly, the ACBD3 KO cells that did get infected seemed to contain more E signal than the WT cells (Fig. 3D). Therefore, we decided to further investigate the levels of viral components in the subpopulation of infected ACBD3 KO cells. Additional fluorescence microscopy analysis revealed that at 16 h after MOI 1 LGTV infection, both E and NS3 were focused as bright foci in the ACBD3 KO cells, and these foci were not observed in the WT cells (Fig. 3F). To quantify this phenomenon, we determined the regions in cells where the NS3 fluorescence intensity was continuously above a set threshold, and the detected area was larger than 10 µm^2^. We then quantified the mean NS3 and E fluorescence intensity within these areas. Notably, the NS3 and E signals in the NS3-positive areas of the ACBD3 KO cells were significantly higher compared with those found in the WT cells (Fig. 3G and H). This shows that although the bulk RNA, viral protein, and virus particle production is reduced as a result of ACBD3 KO, the effects are not uniform across the cell population. Instead, the majority of the cells do not appear to get infected, but the ones that do abnormally accumulate viral components.

**Fig 4:**
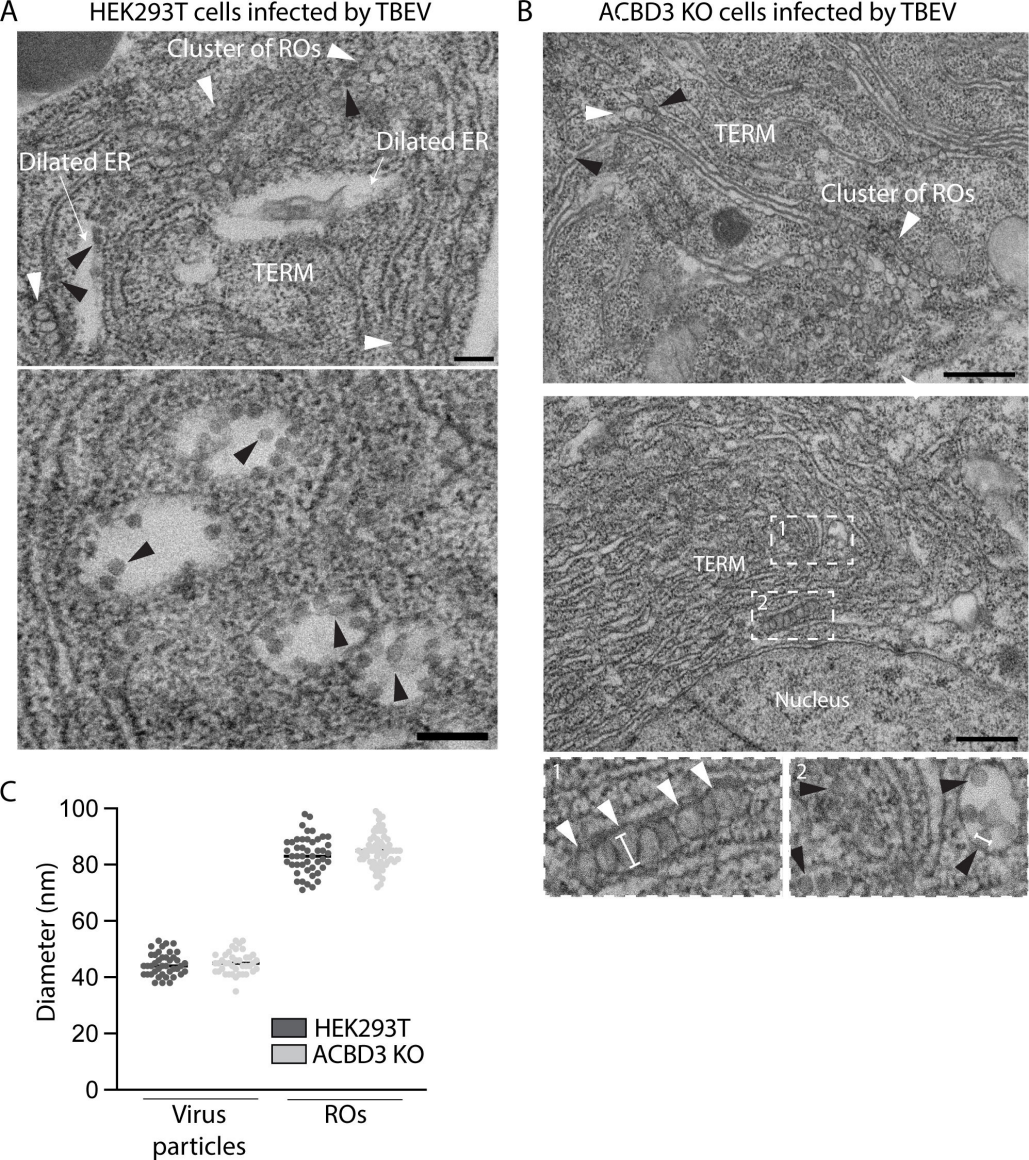
ACBD3 KO does not affect the ultrastructure in infected cells. (**A and B**) Representative electron micrographs of HEK293T (**A**) or ACBD3 KO (**B**) cells infected with TBEV (MOI 1, 24 h.p.i.) shown at two magnifications. The micrographs contain both replication organelles (ROs) (white arrowheads) and viral particles (black arrowheads). Other notable features are indicated. The insets in (**B**) show close-ups of areas 1 and 2. Scale bars (**A**): 200 nm, (**B**): 500 nm. (**C**) Size quantification of identified virus particles and ROs in HEK293T and ACBD3 KO cells. Quantification was performed along the longest axis, as shown by the white lines in (**B**) insets 1 and 2.

To investigate if ACBD3 depletion has effects on the ultrastructure of the TERM, we analyzed WT and ACBD3 KO cells infected by TBEV using electron microscopy (EM). In WT cells, the ER was transformed into tightly packed membrane convolutions with clusters of ROs and typical dilated membrane compartments containing virus particles, as previously shown (Fig. 4A) (9). Similarly, characteristic convoluted membranes containing ROs and virions were observed in the TERM of ACBD3 KO cells (Fig. 4B). However, dilated membrane compartments were much less frequently observed, in agreement with a condensation of the TERM. Quantification of virion and RO diameter did not reveal any significant difference between WT and ACBD3 KO cells (Fig. 4C). These data showed that ROs and virions can still form in the absence of ACBD3, suggesting that ACBD3 is not a core component of either structure. Taken together, these data imply that ACBD3 is involved in the coordination of TERM formation.

### Both RNA replication and virus-like particle secretion are impaired in ACBD3 KO cells

**Fig 5:**
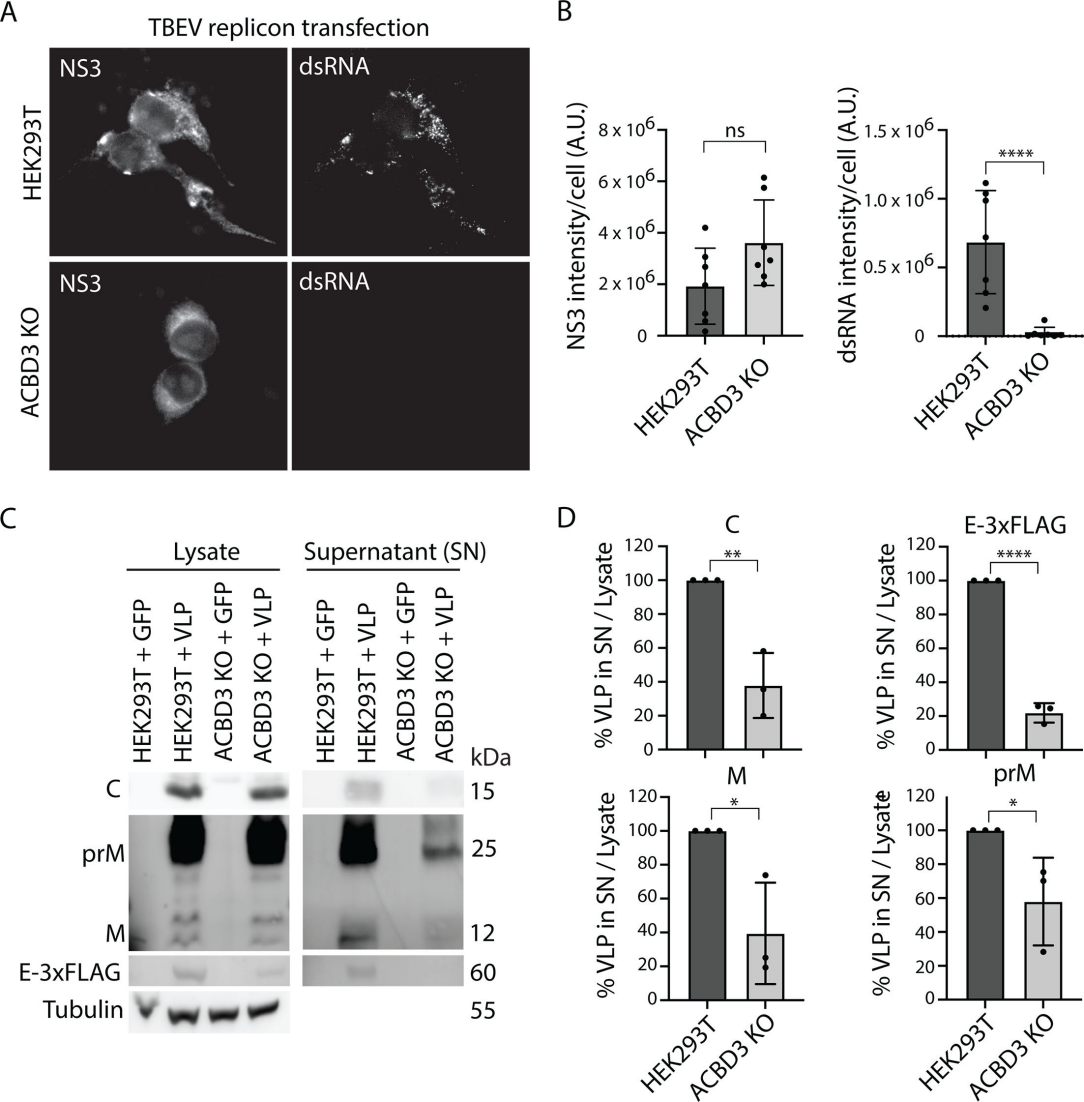
ACBD3 KO reduces replication and VLP secretion. (**A**) Fluorescence micrographs of LGTV-infected (MOI 1, 24 h.p.i.) HEK293T and ACBD3 KO cells transfected with TBEV-replicon for 24 h and stained with anti-NS3 and anti-dsRNA antibodies as indicated. Scale bar, 10 µm. (**B**) Quantification of the total intensity of NS3 and dsRNA stain in TBEV replicon-transfected cells. Mean ± SD of 7 images per condition with 2–12 transfected cells per image from three biological replicates. Unpaired *t*-test. (**C and D**) Immunoblot analysis of VLP secretion in HEK293T cells and ACBD3 KO cells transiently co-transfected with C, prM, and E-3xFLAG (24 hours) using antibodies against C, M/prM, and 3×FLAG. Representative blots are shown in (**C**) and quantification of the densitometric data in (**D**). Data were normalized to HEK293T + VLP control. Mean ± SD of 3 biological replicates. Unpaired *t*-test. ns *P* > 0.05, *P < 0.05, **P < 0.005, ****P < 0.0001 in (**B**) and (**D**).

The uneven effects of ACBD3 KO suggested that it perturbs multiple viral processes. Therefore, we assayed the role of ACBD3 on viral RNA replication and virion production separately. First, we used a TBEV replicon system, which allowed us to focus on effects specific to the NS proteins and the viral RNA (44). In this system, ssRNAs encoding GFP and TBEV NS proteins are transcribed under a pCMV promoter, which results in initial expression of these proteins. If the proteins can perform their functions and replicate the ssRNA, dsRNA will be detected in cells. This subsequently leads to further expression of NS proteins from the positive RNA strand. We used fluorescence microscopy and anti-NS3 antibodies to focus on the successfully transfected WT HEK293T and ACBD3 KO cells at 24 h post-transfection (Fig. 5A). Transfection efficiency was similar in WT and KO cells (WT 4.5% ± 1.3% and KO 2.7% ± 0.7%, *P*-value: 0.27). The NS3 levels did not significantly differ between the WT and ACBD3 KO cells, showing that NS protein expression is not reduced by ACBD3 depletion (Fig. 5A and B). However, dsRNA was hardly detected in the ACBD3 KO cells expressing NS3 (Fig. 5A), and quantification showed that the dsRNA levels were dramatically reduced in comparison to WT HEK293T cells expressing NS3 (Fig. 5B). This shows that although the NS proteins are present, the lack of ACBD3 in cells prevents efficient replication of dsRNA.

Although the RNA replication defects could explain the lower bulk yields of viral RNA, virions, and viral proteins, they should not lead to the accumulation of viral components. As ACBD3 is involved in ER to Golgi trafficking, we investigated if depleting it could impede virion export using a virus-like particle (VLP) secretion assay (42, 45). This allowed us to decouple the genome replication effects of ACBD3 KO from those affecting virion assembly and egress. We transfected ACBD3 KO cells with plasmids expressing C, prM, and E-3×FLAG, which results in the assembly and secretion of VLPs (42). Immunoblot analysis showed that although both cell types produced similar amounts of viral proteins, the secreted fraction was significantly lower in ACBD3 KO cells compared with WT cells (Fig. 5C and D). Together with the earlier microscopy observations, this suggests that ACBD3 is important for the trafficking of virions or viral proteins, presumably by coordinating them in the TERM.

### ACBD3 promotes LGTV and TBEV infections via a PI4KB-independent mechanism

**Fig 6:**
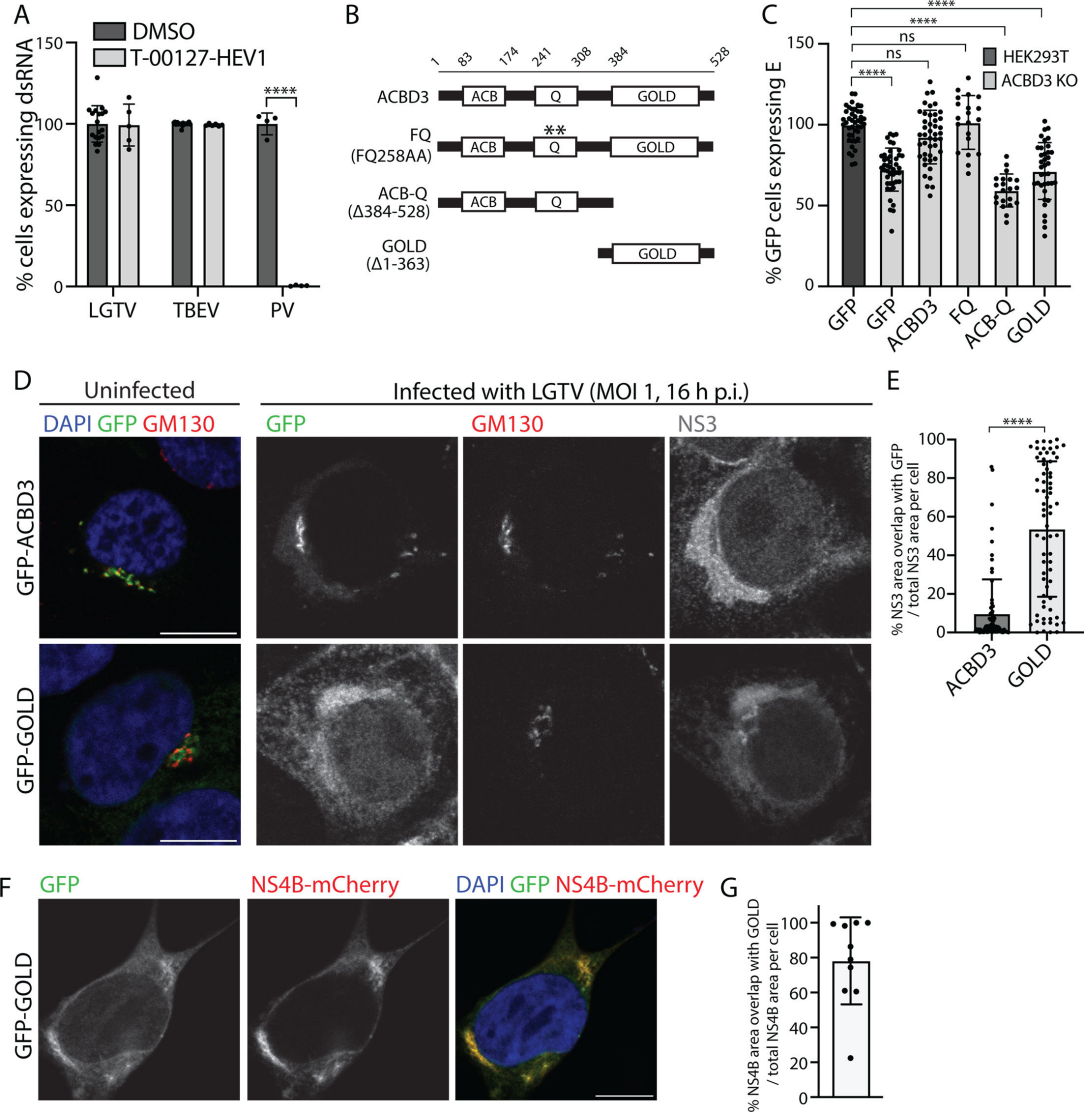
Full-length ACBD3 promotes flavivirus infection independently of PI4KB. (**A**) Quantification of the percentage of A549 cells positive for dsRNA after infection with LGTV, TBEV (24 h.p.i, MOI 0.1), or PV (6 h.p.i., MOI 10) in the absence or presence of T-00127-HEV1 (1.25 µM). Data were normalized to DMSO-treated infected A549 cells. Mean ± SD of at least five biological replicates from three independent experiments, unpaired *t*-test. (**B**) Schematic illustration of ACBD3 domains and the ACBD3 mutants used in the study. (**C**) Quantification of the percentage of GFP-positive HEK293T and ACBD3 KO cells expressing E protein at 24 h after TBEV infection (MOI 0.1) after transfection with different ACBD3 constructs. Data were normalized to GFP-positive HEK293T cells. Mean ± SD of at least 10 biological replicates from two independent experiments. One-way ANOVA with Dunnett multiple tests. (**D**) Confocal fluorescence micrographs of ACBD3 KO cells transiently over-expressing GFP-ACBD3 or GFP-GOLD stained with anti-GM130 and NS3 (only in the infection experiment) antibodies either without infection (left) or 16 h after LGTV infection at MOI 1 (right). Scale bar, 10 µm. (**E**) Quantification of the percentage of NS3 area overlapping with GFP in GFP-ACBD3 or GFP-GOLD-transfected cells. Data are presented in scatter plots with mean ± SD. Number of NS3 areas analyzed, GFP-ACBD3 (*n* = 130), GFP-GOLD (*n* = 179). Mann-Whitney test. (**F**) Confocal fluorescence micrographs of ACBD3 KO cells transiently expressing NS4B-mCherry and GFP-GOLD. Scale bar, 10 µm. (**G**) Quantification of the percentage of the fluorescence NS4B area overlapping with the fluorescence of GFP-GOLD. Data are presented in a scatter plot with mean ± SD. The number of cells analyzed is indicated. ns *P* > 0.05, ****P < 0.0001. **P < 0.005, ****P < 0.0001 in (**A**), (**C**), (**E**), and (**G**).

ACBD3 has a known pro-enteroviral role. During enterovirus infection, ACBD3 recruits PI4KB to RNA replication sites to promote the synthesis of the phosphatidylinositol-4-phosphate (PI4P) lipids that are critical for the enteroviral RNA polymerase (46). Therefore, we investigated if impaired PI4KB recruitment could explain the effects of ACBD3 absence by using the PI4KB inhibitor T-00127-HEV1 (46). The treatment had no effect on cell viability up to 5 µM, and as previously reported, it reduced poliovirus (PV) infection in a dose-dependent manner with near-complete inhibition observed at 1.25 µM (Fig. S4A and B). However, at 1.25 µM, the compound did not inhibit LGTV or TBEV replication in A549 cells, showing that PI4KB recruitment is not the proviral mechanism for ACBD3 in flavivirus infection (Fig. 6A).

### Full-length ACBD3 is needed to promote flavivirus infection

ACBD3 is a multi-domain protein composed of an ACB domain (ACB) that binds acyl-CoA, a Q domain that interacts with PI4KB, and a Golgi dynamics domain (GOLD) that facilitates various membrane and protein interactions (47). To investigate which domain(s) are needed to promote TBEV infection, we transfected ACBD3 KO cells with constructs expressing GFP-tagged WT or mutant ACBD3 (Fig. 6B). Next, we infected the cells with TBEV and quantified the fraction of transfected cells (expressing GFP) that was also infected (expressing E) with a plate reader. Transfection with WT ACBD3 rescued the infection, but transfection with either the GOLD domain on its own or the ACB-Q construct lacking the GOLD domain did not (Fig. 6C). Interestingly, productive infection was also restored by complementation with the FQ mutant that has a non-functional PI4KB binding site (46), further confirming that ACBD3 does not promote flavivirus infection via PI4KB recruitment (Fig. 6C).

As ACBD3 seems to have a role in the proper trafficking of viral components, and it colocalizes with ERES-Golgi markers in infected and healthy cells, we investigated which ACBD3 domain(s) is needed to correctly localize it at the TERM. Fluorescent microscopy analysis of GFP-tagged truncated protein constructs expressed in ACBD3 KO cells showed that GFP-ACBD3 and GFP-GOLD co-localized with the *cis*-Golgi marker GM130 (Fig. 6D). However, GFP-ACB-Q was primarily detected in the cytosol, showing that GOLD mediates membrane targeting (Fig. S4C). During infection, GFP-ACBD3 co-localized with GM130, and GFP-ACB-Q was detected throughout the cytosol similarly as in healthy cells. However, GFP-GOLD was detected over the whole NS3-positive TERM (Fig. 6D; Fig. S4C), with a significantly larger area of overlap with NS3 compared with full-length ACBD3 (Fig. 6E). Furthermore, the expression of NS4B-mCherry was enough to change the GFP-GOLD localization to the entire ER where it colocalized with NS4B-mCherry (Fig. 6F and G). These data show that the GOLD domain is likely responsible for the proximity of NS4B and ACBD3 during infection. However, the GOLD domain alone is not enough to rescue infection. Instead, the full-length protein that localizes at TERM-Golgi contacts (GM130-positive areas) during infection is needed. Together, this suggests that ACBD3 facilitates flavivirus infection by bringing the NS4B and TERM-Golgi contact sites in close contact, either by direct or by indirect interactions.

## DISCUSSION

The general characteristics of the flavivirus maturation and egress pathway were elucidated decades ago by groundbreaking biochemical and structural work on TBEV and dengue viruses (30, 45, 48, 49). However, the model was built on the assumption that the ER and the Golgi function similarly in infected and healthy cells. Instead, electron and light microscopy observations from multiple flavivirus species show that both the ER and the Golgi, as well as the cellular lipidome, are extensively remodeled during infection (6, 7, 11–13, 50–53). Traditionally, this has been seen as a way to facilitate RNA replication, for example, by RO generation. However, it is likely that from the very early stages of the infection, the cellular membranes are transformed in a coordinated manner that allows for the synthesis of RNA, its efficient packaging into assembling virions, and their export from the cell. Similarly, virion maturation and secretion are probably more complex than thought earlier, especially since recent work has demonstrated that also in uninfected cells, ER-Golgi trafficking includes previously undescribed mechanisms (54–57).

Our APEX2 screen of NS4B-proximal host factors identified 173 candidate proteins, with 20 of the hits implicated in ER-to-Golgi transport. Furthermore, four of the five top hits (ACBD3, TFG, SEC23IP, and KTN1) are associated with the ERES (34–40). We followed up on ACBD3 since knocking it out had the greatest effect on LGTV infection, and it affected TBEV and WNV as well. ACBD3 depletion led to a reduction in the number of infected cells, bulk RNA replication (as early as 8 h.p.i.), viral protein synthesis, and virion production. Additionally, ACBD3 KO abolished dsRNA production in a TBEV replicon assay without affecting viral protein translation. Therefore, we initially assumed that ACBD3 would take part in RO generation, a canonical NS4B function. However, ACBD3 did not colocalize with dsRNA in infected cells, and it was instead found at ERES-Golgi contact sites (positive for SEC23IP and GM130) alongside NS4B. EM observations confirmed that ACBD3 KO did not abolish RO formation. Therefore, ACBD3 and NS4B work together to promote infection with a mechanism outside of RO generation. Our data suggest that this is mediated by either direct or indirect contacts between NS4B and the GOLD domains. When we investigated the effects of ACBD3 KO on the cells that did get infected, we noticed the accumulation of viral components in the TERM, which is consistent with trafficking defects. This was confirmed with the use of an infection-free VLP secretion assay, which showed that ACBD3 depletion affects VLP egress independent of any RNA replication effects. The VLP secretion assay does not differentiate between general or virus-specific export defects. Therefore, it will be important to characterize the extent of the effects of ACBD3 KO to understand if this protein mediates an export pathway that only viral particles use or if the virions exploit a general export mechanism that ACBD3 is a part of.

TERM is the central scaffolding upon which flavivirus production occurs. It needs to be generated in a coordinated way not only to facilitate RNA replication but also to ensure that the newly synthesized genomes are efficiently packaged and that the resulting particles are trafficked out of the cell via the correct chemical environments that lead to virion maturation. The non-uniform results of ACBD3 are consistent with a possible role in TERM generation. TERM defects could result in difficulties in the early stages of infection, leading to a higher likelihood of abortive infection. Additionally, even if this initial hurdle is passed, a defective TERM could result in the incorrect localization of viral structural and nonstructural proteins, leading to their accumulation. Therefore, it is tempting to speculate that tick-borne flaviviruses could utilize the ACBD3-NS4B affinity to recruit NS4B at ERES-Golgi sites (or vice versa) during TERM generation. It is possible that the formation of these contacts works as a checkpoint for RO generation to ensure that new genomes are only synthesized near enough ERES-Golgi contact sites to ensure that the subsequently assembled virions are correctly trafficked.

Interestingly, we show that despite ACBD3’s well-characterized role in enterovirus infection, it promotes flavivirus infection with a different mechanism not related to PI4KB recruitment and PI4P synthesis (58, 59). This is not surprising considering that unlike in the ER-centric flavivirus replication cycle, enteroviral RNA synthesis and assembly occur on cytoplasmic membrane vesicles, and virion release is mediated via autophagosomes (54, 55, 60). However, it is intriguing that ACBD3 is hijacked by both distantly related viral taxa.

**Fig 7:**
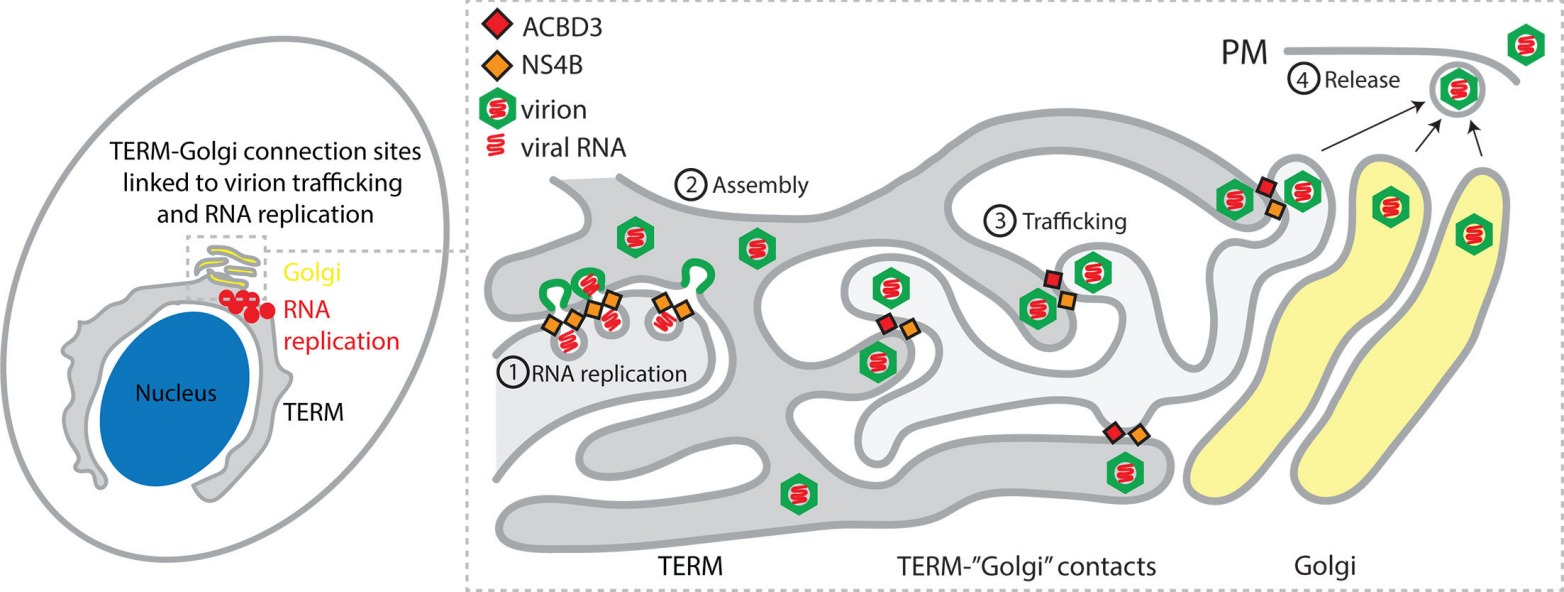
Model of TERM and ACBD3 function in flavivirus infection. Schematic illustration of the proposed roles of NS4B and ACBD3 in promoting connection sites between the TERM and the cis-Golgi in order to promote secretion of virions. Viral RNA genomes are replicated in TERM-derived ROs, which contain NS4B and other viral and host proteins (1), and the genomes interact with viral structural proteins to form immature virions that bud into the lumen of the TERM (2). The virions are trafficked to the ERES-Golgi contact sites that have been modified by NS4B and host proteins to facilitate the transport of large viral cargoes to the Golgi (3). In the Golgi, it is unknown if the virions are trafficked through the Golgi like canonical exocytic cargoes, or if they are transported straight to the PM for release (4).

Based on our results, we propose a model where ACBD3 recruits NS4B at ERES-Golgi contact sites. This occurs in the early stages of the infection to ensure that RNA replication and subsequent virion assembly occur in close proximity to the sites where the TERM connects to the *cis*-Golgi to facilitate the efficient transfer of virions to the Golgi (Fig. 7). The ERES are likely modified during the process to enable controlled transport of the relatively large virions while, at the same time, ensuring that viral proteins remain concentrated in the TERM. ACBD3 remodels ERES-Golgi contacts to mediate Golgi transport of the STING receptor during bacterial infection, suggesting that it is involved in the process during flavivirus infection as well (39, 61). Analysis of trafficking dynamics in healthy live cells has demonstrated that bidirectional transport portals are formed between the ERES and arrival sites at the Golgi (56). Additionally, ultrastructural analysis has revealed the presence of a tubular membrane network extending from the COPII-positive ER to the COPI-positive *cis*-Golgi (57). Whether the TBEV virion trafficking process involves the fusion of ER and Golgi membranes into tubular portals or if it is vesicle-based remains to be elucidated. In the conventional secretory pathway, ACBD3 has been proposed to control the retrograde trafficking of KDEL receptors (KDELR) from the Golgi to ER. ACBD3 forms a complex with KDELR, and ACBD3 depletion resulted in KDELR accumulation at the ER and the disruption of KDELR cycling (62, 63). Interestingly, both prM and E on the immature DENV and JEV virion surfaces have been shown to bind to KDELR to facilitate particle egress (64, 65). Therefore, it is likely that KDELR-ACBD3 contacts take part in flavivirus particle trafficking. However, as ACBD3 KO had no effect on JEV RNA replication, it is possible that tick-borne flavivirus species have a stronger coupling between the RNA synthesis and particle egress sites, whereas mosquito-borne species do not require the sites to be coordinated for genome replication.

In summary, we identified ACBD3 as a proviral host factor for tick-borne flaviviruses. ACBD3 associates with NS4B to facilitate TERM generation, both for RNA replication as well as virion export. Although the vast rearrangement of host membranes is critical for productive flavivirus infection, the details of the process remain understudied, and our results serve as a starting point to elucidate the role of ERES-Golgi contact sites in flaviviruses.

## MATERIALS AND METHODS

### Cell lines and virus strains

HEK293T cells, ACBD3 KO cells, and A549 cells were cultured in Dulbecco’s modified Eagle medium (DMEM) supplemented with 10% FBS (Gibco, Thermo Fisher Scientific) at 37°C, 5% CO_2_. ACBD3 KO cells were a gift from Frank van Kuppeveld (43). LGTV strain TP21 (kindly provided by Gerhard Dobler, Bundeswehr Institute of Microbiology, Munich, Germany) was propagated in A549 MAVS KO (Abcam, cat no. ab282354) cells. TBEV strain Töro-2003 (66) was propagated in Vero B4 cells. Additional virus strains (JEV, WNV, PV) used in the study are listed in Table 1. Viral titers of flaviviruses were determined by focus-forming assay (9), and PV titer was determined by plaque assay (described in the following section).

**TABLE 1:** Virus strains used in the study

Virus	Strain and isolation	Cell line for propagation	Source
LGTV	TP21	A549 MAVS KO	Gift from Gerhard Dobler, Bundeswehr Institute of Microbiology, Munich, Germany
TBEV	Torö−2003	Vero B4	(66)
JEV	Nakayama	Vero B4	Gift from Sirkka Vene, Public Health Agency of Sweden, Stockholm, Sweden
WNV	Isolated in 2003 in Israel WNV_0304 h_ISR00	Vero B4	Gift from Sirkka Vene, Public Health Agency of Sweden, Stockholm, Sweden
PV	Type 1 Mahoney	A549	Gift from George Belov, University of Maryland, Maryland, United States of America

### Focus-forming assay

Vero B4 cells in 96-well plates were infected with serially diluted virus samples (supernatant of infected culture) in DMEM for 2 h at 37°C, 5% CO_2_. The inocula were then replaced with DMEM supplemented with 2% FBS and 1.2% Avicel (FMC BioPolymer). After 48 h.p.i., the cells were fixed in 4% formaldehyde for 20 min and then permeabilized with 0.5% Triton X-100 in PBS. The cells were then incubated with anti-E antibodies for 1 h at RT. The cells were then incubated with horseradish peroxidase-conjugated secondary antibodies for 1 h at RT. Chromogenic peroxidase substrate Trueblue (KPL, Seracare) was added to the plates for focus visualization. The plates were imaged with a Cytation 5 plate imager (Biotek), and the foci were counted manually. The viral titer was expressed as focus-forming units per mL (FFU/mL).

### Plaque assay

Confluent cells in 6-well plates were infected with serially diluted virus samples in minimum essential medium (MEM) for 1 h at 37°C, 5% CO_2_. The inocula were then replaced with MEM supplemented with 2% FBS and 0.3% agarose. After 48 h.p.i., the cells were fixed with 5% formaldehyde for 24 h. The cells were then rinsed with water and stained with 3% crystal violet for plaque visualization. The stained cells were then washed with water. The plaques were counted manually. The viral titer was expressed as plaque-forming units per mL (PFU/mL).

### Antibodies, probes, plasmids, and replicon

The antibodies and probes used in the study are listed in Table 2. The plasmids used in the study are listed in Table 3.

**TABLE 2:** Antibodies and probes used in the study^*a*^

Antibody/probe	Dilution	Source	Identifier
Anti-CANX	1:50,000 (IB), 1:1,000 (IF)	Abcam	Cat# ab22595
Anti-ACBD3	1:2,000 (IB), 1:1,000 (IF)	Atlas Antibodies	Cat# HPA015594
Anti-GM130	1:300 (IF)	BD Transduction	Cat# 610823
Anti-SMARCA1	1:500 (IF)	Atlas Antibodies	Cat# HPA064712
Anti-SEC23IP	1:500 (IF)	Atlas Antibodies	Cat# HPA043305
Anti-KTN1	1:1,000 (IB)	Atlas Antibodies	Cat# HPA003178
Anti-TFG	1:1,000 (IB)	Atlas Antibodies	Cat# HPA019473
Anti-GAPDH	1:5,000 (IB)	Merck Millipore	Cat# MAB374
Anti-Tubulin	1:4,000 (IB)	Abcam	Cat# ab6046
Anti-dsRNA clone J2	1:1,000 (IF)	Scicons, Nordic MUbio	Cat# 10010500
Anti-NS3	1:1,000 (IF), 1:5,000 (IB)	(67)	
Anti-C	1:1,000 (IB)	(42)	
Anti-M	1:1,000 (IB)	(68)	
Anti-E clone 1786.3	1:1,000 (IF)	(69)	
Anti-E clone 5G5^*b*^	1:1,000 (IB)	BEI Resources (70)	Cat# NR-40318
Anti-FLAG clone M2	1:1,000 (IB)	Sigma	Cat# F1804
Anti-rabbit IgG 680RD or 800CW	1:20,000 (IB)	Li-Cor Biosciences	Cat# 926–68071, 926–32213
Anti-mouse IgG 680RD or 800CW	1:20,000 (IB)	Li-Cor Biosciences	Cat# 926–68070, 926–32210
Anti-chicken IgY 800CW	1:10,000 (IB)	Li-Cor Biosciences	Cat# 926–32218
Anti-rabbit AlexaFluor 488, 568, 647	1:1,000 (IF)	Invitrogen	Cat# A11034, A21069, A21246
Anti-mouse AlexaFluor 568, 647	1:1,000 (IF)	Invitrogen	Cat# A11031, A21246
Anti-chicken AlexaFluor 568, 647	1:1,000 (IF)	Invitrogen	Cat# A11041, A21449
Streptavidin 680RD	1:10,000 (IB)	Li-Cor Biosciences	Cat# 68079

^*a*^ IB, immunoblot; IF, immunofluorescence.

^*b*^ The anti-E clone 5G5 was obtained from the Joel M. Dalrymple - Clarence J. Peters USAMRIID Antibody Collection through BEI Resources, NIAID, NIH: Monoclonal Anti-Langat Virus Envelope Glycoprotein (E), Clone 5G5 (produced *in vitro*), NR-40318.

**TABLE 3:** Plasmids used in the study

Plasmid	Source	Identifier
pOG44 (Flp recombinase)	Invitrogen, Thermo Fisher Scientific	Cat # V600520
pcDNA5/FRT/TO/2K-NS4B-3xFLAG-APEX2 (NS4B-APEX2)	This study	
TBEV subgenomic replicon	Gift from Magnus Johansson (44)	
pcDNA3.1–2K-NS4B-EGFP (NS4B-GFP)^*a*^	This study	
pcDNA3.1–2K-NS4B-mCherry (NS4B-mCherry)^*a*^	This study	
pI.18-C-3xFLAG	(42)	
pI.18-prM-3xFLAG	(42)	
pI.18-E-3xFLAG	(42)	
pEGFP-SMARCA1	Gift from David Picketts (71)	
pEGFP-KTN1	Gift from Hanry Yu (72)	
pEGFP-TFG	Gift from Marc Servant (73)	
pEGFP-SEC23IP	Gift from Mitsugo Tagaya (74)	
pEGFP-ACBD3 (WT)	Gift from Carolyn Machamer (75)	Addgene plasmid #180633
pEGFP-ACBD3-1-383 (ACB-Q)^*b*^	This study	
pEGFP-ACBD3-363-528 (GOLD)	Gift from Frank van Kuppeveld (58)	
pEGFP-ACBD3-FQ258AA (FQ)	Gift from Frank van Kuppeveld (58)	
pSpCas9(BB)-GFP	Gift from Feng Zhang (76)	Addgene plasmid #48138

^*a*^ Plasmids pcDNA3.1–2K-NS4B-EGFP or -mCherry were generated similar to pcDNA5/FRT/TO/2K-NS4B-3xFLAG-APEX2 (described in the following section) and ligated into pcDNA3.1 with either EGFP or mCherry tag.

^*b*^ Plasmid pEGFP-ACBD3-1-383 (ACB-Q) was generated by amplifying the coding sequence of ACB and Q domain (amino acid 1–383) from the template pEGFP-ACBD3 (WT) using primers containing restriction sites BglII (5’) and EcoRI (3’) and then ligated into vector pEGFP-C1.

### Generation of the NS4B-3xFLAG-APEX2 plasmid and the NS4B-APEX2 cell line

The coding sequence of APEX2 was PCR-amplified from APEX2 plasmid (a gift from Alice Ting, Addgene plasmid #72480, [77]) using primers containing restriction sites NotI (5’) and XhoI (3’). The amplified 5’ NotI-APEX2-XhoI 3’ fragments were digested and ligated into the vector pcDNA5/FRT/TO (Invitrogen, Thermo Fisher Scientific). Synthetic oligonucleotides encoding 3×FLAG were then ligated to the 5’ position of APEX2 sequence using the NEBuilder HiFi DNA assembly kit (New England Biolabs) to form the final vector pcDNA5/FRT/TO/3xFLAG-APEX2.

Next, the coding sequence of 2K-NS4B was PCR-amplified from TBEV subgenomic DNA replicons (44) using specific primers containing restriction site KpnI (5’) and NotI (3’). The sequence of 2K was included to ensure the correct topology of NS4B at the ER membrane. The amplified 5’ KpnI-2K-NS4B-NotI 3’ fragments were digested and ligated into the vector pcDNA5/FRT/TO/3xFLAG-APEX2 to form the plasmid NS4B-3xFLAG-APEX2 for the NS4B-APEX2 cell line.

Flp-In T-Rex HEK293T cells (Invitrogen, Thermo Fisher Scientific) were co-transfected with plasmids pOG44 (Invitrogen, Thermo Fisher Scientific) and pcDNA5/FRT/TO/2K-NS4B-3xFLAG-APEX2 in 9:1 (wt/wt) ratio using GeneJuice transfecting agent (Sigma-Merck) according to the manufacturer’s instructions. After 2 days of incubation, the cells were selected by 200 µg/mL hygromycin B (Invivogen) for 7 days. The cell line was maintained as above in a medium containing 5 µg/mL of blasticidin (Invivogen) and 100 µg/mL of hygromycin B (Invivogen). Expression of NS4B-APEX2 protein was induced by incubating the cells with 1 ng/mL of doxycycline (Dox) (Invitrogen, Thermo Fisher Scientific) for 16 h.

### APEX2 biotinylation and pulldown

Confluent NS4B-APEX2 cells were incubated in 10 cm dishes with media containing 1 ng/mL Dox for 16 h to induce the expression of NS4B-APEX2 proteins. In case of LGTV infection, the cells were infected with LGTV (MOI 10) for 2 h prior to Dox induction. APEX2-biotinylation was performed as previously published (33). Briefly, the cells were treated with 50 µM biotin-phenol (Iris Biotech) for 30 min at 37°C. The cells were then incubated with 1 mM H_2_O_2_ for 1 min at room temperature (RT) to trigger biotinylation. The reaction was then quenched by washing the cells three times with ice-cold PBS buffer containing 5 mM Trolox (Sigma-Merck), 10 mM sodium ascorbate, and 10 mM sodium azide. For pulldown of biotinylated proteins, the cells were lysed with ice-cold radioimmunoprecipitation assay buffer (RIPA) buffer (25 mM HEPES pH 7.5, 150 mM NaCl, 1 mM EDTA, 1 mM EGTA, 1% NP-40, 1% sodium deoxycholate, and 0.1% SDS) containing 5 mM Trolox, 10 mM sodium ascorbate, and 10 mM sodium azide, protease, and phosphatase inhibitors (Roche). The lysates were centrifuged at 20,000 × *g* for 10 min at 4°C. The cleared supernatants were incubated with 50 µL of neutravidin agarose bead slurry (Pierce, Thermo Fisher Scientific) at 4°C overnight. The beads were then washed once with each of the following buffers: RIPA buffer, 1 M KCl in H_2_O, 0.1 M Na_2_CO_3_ in H_2_O, 2 M urea in 10 mM Tris HCl, pH 7.4, and final 2% SDS in 150 mM NaCl with 25 mM HEPES pH 7.5. The washed beads were then incubated with 50 µL of 2× Laemmli buffer (without bromophenol blue and glycerol) containing 50 mM dithiothreitol (DTT) and 5 mM biotin for 10 min at 75°C to elute the bound proteins. The samples were either analyzed with immunoblotting or further processed for quantitative proteomic analysis.

### Quantitative proteomic analysis

#### Sample preparation

The APEX2 biotinylation pulldown samples (mock, NS4B-APEX2, and NS4B-APEX2 with LGTV infection in triplicate, 9 samples in total) were processed using the modified filter-aided sample preparation (FASP) method (78, 79). In short, eluates were reduced with 100 mM DTT at 60°C for 30 min, transferred to Microcon-30kDa Centrifugal Filter Units (Merck), washed several times with 8 M urea and once with digestion buffer (DB, 50 mM TEAB, 0.5% sodium deoxycholate [SDC]) prior to alkylation with 10 mM methyl methanethiosulfonate in DB for 30 min at RT. Samples were digested with trypsin (0.3 µg Pierce MS grade Trypsin, Thermo Fisher Scientific) at 37°C overnight, and an additional portion of trypsin (0.3 µg) was added: the samples were incubated for another 2 h. The samples were combined into one tandem mass tag (TMT) set, and SDC was removed by acidification with 10% trifluoroacetic acid (TFA). The TMT set was further purified using High Protein and Peptide Recovery Detergent Removal Spin Column and Pierce peptide desalting spin columns (both Thermo Fischer Scientific) according to the manufacturer’s instructions. Basic reverse phase peptide separation was performed using a Dionex Ultimate 3000 UPLC system (Thermo Fischer Scientific) and a reversed-phase XBridge BEH C18 column (3.5 µm, 3.0 × 150 mm, Waters Corporation) with a gradient from 3% to 100% acetonitrile in 10 mM ammonium formate at pH 10.00 over 27 min at a flow of 400 µL/min. The 10 fractions were dried and reconstituted in 3% acetonitrile, 0.1% TFA.

#### LC-MS analysis

The fractions were analyzed on an Orbitrap Fusion Tribrid mass spectrometer interfaced with nLC 1200 liquid chromatography system (both Thermo Fisher Scientific). Peptides were trapped on an Acclaim Pepmap 100 C18 trap column (100 µm × 2 cm, particle size 5 µm, Thermo Fischer Scientific) and separated on an in-house constructed analytical column (350 × 0.075 mm I.D.) packed with 3 µm Reprosil-Pur C18-AQ particles (Dr. Maisch, Germany) using a gradient from 3% to 80% acetonitrile in 0.2% formic acid over 90 min at a flow of 300 nL/min. Precursor ion mass spectra were acquired at 120,000 resolution, scan range 375–1375, and a maximum injection time of 50 ms. MS2 analysis was performed in a data-dependent mode, where the most intense doubly or multiply charged precursors were isolated in the quadrupole with a 0.7 m/z isolation window and dynamic exclusion within 10 ppm for 60 s. The isolated precursors were fragmented by collision-induced dissociation (CID) at 35% collision energy with the maximum injection time of 50 ms for 3 s (“top speed” setting) and detected in the ion trap, followed by multinotch (simultaneous) isolation of the top 10 MS2 fragment ions within the m/z range 400–1200, fragmentation (MS3) by higher-energy collision dissociation (HCD) at 65% collision energy and detection in the Orbitrap at 50,000 resolution m/z range 100–500, and maximum injection time 105 ms.

#### Database matching and quantitative analysis

The raw files were merged for identification and relative quantification using Proteome Discoverer version 2.4 (Thermo Fisher Scientific). The search was against SWISS-PROT *Homo sapiens* (80*)* and a custom database containing LGTV polyprotein and background proteins (using Mascot 2.5 [Matrix Science]) as a search engine with precursor mass tolerance of 5 ppm and fragment mass tolerance of 0.6 Da. Tryptic peptides were accepted with zero missed cleavages; variable modifications of methionine oxidation and fixed cysteine alkylation and TMT-label modifications of N-terminal and lysine were selected. Percolator was used for PSM validation with the strict FDR threshold of 1%. TMT reporter ions were identified with 3 milli-mass units mass tolerance in the MS3 HCD spectra, and no normalization was applied. Only the quantitative results for the unique peptide sequences with the minimum SPS match % of 55 and the average S/N above 10 were taken into account for the protein quantification. The quantified proteins were filtered at 1% FDR and grouped by sharing the same sequences to minimize redundancy.

### CRISPR-Cas9 knockdown and flow cytometry

The guide RNA (listed in Table 4) was designed with the CRISPR design tool at Benchling (www.benchling.com) and cloned into vector pSpCas9(BB)-GFP (a gift from Feng Zhang, Addgene plasmid #48138) as described (76, 81). The cloned plasmids were transfected into HEK293T cells using GeneJuice transfecting agent (Merck Millipore). Forty-eight hours after transfection, the cells were re-seeded and transfected again for 48 h. Following this, the cells were then re-seeded into 12-well plates and infected with LGTV at MOI 1 for 2 h at 37°C, 5% CO_2_. The inocula were then replaced with DMEM supplemented with 2% FBS. After 24 h.p.i., the cells were fixed in 4% formaldehyde for 20 min and then washed three times with FACS buffer (20 mM EDTA, 2% FBS, 0.02% sodium azide in PBS) containing 0.5% Triton X-100. The cells were then incubated with anti-E antibodies (clone 1786.3) (69) in FACS buffer for 1 h at RT. Then, the cells were incubated with secondary antibodies AlexaFluor 647 for 1 h at RT. The cells were then analyzed with a BD Accuri C6 flow cytometer (BD Biosciences).

**TABLE 4:** Guide RNA used in the study

Gene target		Guided RNA sequence (5’ to 3’)	Source
SMARCA1	1	CACCGCGTGGCCGCGGTGGCTTCGG	This study
	2	CACCGAAAGTTTCACTGGCGATACC	This study
	3	CACCGATGAGACGGAATACACGTAC	This study
KTN1	1	CACCGAACTACGCCAGGATTATGCT	This study
	2	CACCGTCGGTGTAAGCAGTTAACCC	This study
	3	CACCGACCACTTCCCTGTATTGGCT	This study
ACBD3	1	CACCGAGAGGAAAGGCTTCGACGGG	This study
	2	CACCGCGCTGGGGTTTCGGCCTGG	This study
	3	CACCGAGAAGAAGAAAGGCTTCGGT	This study
TFG	1	CACCGGAGGACGGTTACGCGCATA	This study
	2	CACCGTACCAGCAACAGGCCGGCTA	This study
	3	CACCGAAGTTCTATCAGTTCTCGA	This study
SEC23IP	1	CACCGTACAGCGTCTCACTTCGGC	This study
	2	CACCGAATGGGGCACCACGCAAGA	This study
	3	CACCGAAGTAAGTTAGTGCCCGATG	This study
GBF1	1	CACCGATGGATTACGTCAATCCCCG	(42)
	2	CACCGACACGACCGCCATAACTCAG	(42)
	3	CACCGACAGTGATTGACAGCACCG	(42)

### Immunofluorescence staining

Cells on cover glasses were fixed with 4% formaldehyde for 20 min at RT and then rinsed with PBS. The fixed cells were then permeabilized with 0.1% Triton X-100 in PBS for 10 min at RT and then rinsed with PBS. The cells were then blocked with 2% BSA in PBS containing 0.05% Tween-20 (PBS-T) for 1 h at RT. The cells were then stained with specific primary antibodies and secondary fluorescent antibodies and DAPI diluted in blocking buffer for 1 h each. The fluorescent images were acquired by a Leica SP8 confocal microscope with a HC PL APO 63 ×/1.4 oil CS2 objective (Leica). For SIM, fluorescent images were acquired using a Zeiss Elyra 7 microscope with Lattice-SIM^2^ with a Plan-Apochromat 63 ×/1.4 oil objective (Zeiss).

### Quantification of confocal fluorescence microscopy images

Confocal fluorescence images were analyzed using ImageJ Fiji software (82). NS3 regions in cells were determined by setting the fluorescent intensity threshold to 70. The NS3 regions of size >10 µm^2^ were identified using the “Analyze Particles” function of ImageJ Fiji. The mean fluorescence intensities of NS3, E, or dsRNA within the identified NS3 regions were then measured. For the analysis of the fluorescence overlap, images were thresholded to generate a pixel area map of each fluorescent signal. Overlapping pixels in between different fluorescent signals were identified using the “Image Calculator” function of ImageJ Fiji. Manders’ coefficient of protein colocalization was calculated using the JACoP function of the PTBIOP plugin of ImageJ Fiji software (83).

### Immunoblot analysis

Cells were lysed in 2× Laemmli buffer (Bio-Rad) containing 100 mM DTT and heated for 10 min at 95°C. The cell lysate was then separated by electrophoresis in a 10% polyacrylamide gel and transferred to a polyvinylidene difluoride (PVDF) membrane (Merck Millipore). The membrane was blocked with 5% milk in phosphate-buffered saline with Tween 20 (PBS-T). The blocked membrane was then incubated with primary antibodies for 1 hour at RT or 4°C overnight. The membrane was then incubated with secondary fluorescent antibodies (Li-Cor Biosciences) and detected with Odyssey Fc imager (Li-Cor Biosciences) and analyzed with Image Studio software (Li-Cor Biosciences).

### Virus infection quantification using plate imager

HEK293T cells and ACBD3 KO cells were cultured in 96-well plates (Greiner Bio-one) and infected with indicated flaviviruses for 2 h at 37°C, 5% CO_2_. MOI of the viruses used is described in the figure legends. The inocula were then replaced with DMEM supplemented with 2% FBS. After 24 h.p.i., the cells were fixed in 4% formaldehyde for 20 min and then permeabilized with 0.5% Triton X-100 in PBS. The cells were then incubated with either anti-E antibodies or anti-dsRNA antibodies (specified in the figure legends) for 1 h at RT. Then, the cells were incubated with secondary antibodies AlexaFluor 647 and DAPI for 1 h at RT. The plates were then analyzed with a Cytation 5 plate imager with Gen5 software (Biotek) to quantify the number of either E protein or dsRNA positive cells.

### PI4KB inhibition assay

A549 cells were cultured in 96-well plates (Greiner Bio-one) and infected with indicated flaviviruses at MOI 0.1 for 2 h at 37°C, 5% CO_2_ or PV at MOI 10 for 1 h at 37°C, 5% CO_2_. The inocula were then replaced with DMEM supplemented with 2% FBS. PI4KB inhibitor T-00127-HEV1 (Calbiochem #538001, CAS 900874–91-1) or DMSO control was added to the media. After 24 h.p.i. (6 h.p.i. for PV), the cells were fixed in 4% formaldehyde for 20 min and then permeabilized with 0.5% Triton X-100 in PBS. The cells were then incubated with anti-dsRNA antibodies for 1 h at RT. Then the cells were incubated with secondary antibodies AlexaFluor 647 and DAPI for 1 h at RT. The plates were then analyzed with a Cytation 5 plate imager with Gen5 software (Biotek) to quantify the number of E protein-positive cells.

### Rescue assay with ACBD3 mutants

HEK293T cells and ACBD3 KO cells were transiently transfected with either GFP or the indicated GFP-ACBD3 mutants for 24 h. The transfected cells were then infected with TBEV at MOI 0.1 for 2 h at 37°C, 5% CO_2_. The inocula were then replaced with DMEM supplemented with 2% FBS. After 24 h.p.i., the cells were fixed in 4% formaldehyde for 20 min and then permeabilized with 0.5% Triton X-100 in PBS. The cells were then incubated with anti-E antibodies for 1 h at RT. Then, the cells were incubated with secondary antibodies AlexaFluor 647 and DAPI for 1 hour at RT. The plates were then analyzed with a Cytation 5 plate imager with Gen5 software (Biotek) to quantify the number of GFP-positive and E protein-positive cells.

### Reverse-transcription quantitative PCR (RT-qPCR)

Total RNA of infected cells was extracted using the NucleoSpin RNA Plus kit (Macherey-Nagel) and eluted in 60 µL RNase-free H_2_O according to the manufacturer’s instructions. Ten microliters of total RNA extract were used for cDNA synthesis using the High Capacity cDNA Reverse Transcription kit (Thermo Fisher Scientific) according to the manufacturer’s instructions. LGTV RNA was quantified using qPCRBIO probe mix Hi-ROX (PCR Biosystems) and primers recognizing NS3, forward primer 5′-AACGGAGCCATAGCCAGTGA-3′, reverse primer 5′-AACCCGTCCCGCCACTC-3′ and probe FAM-AGAGACAGATCCCTGATGG-BHQ, TBEV RNA was quantified using the following primers and probe, 5´- GGGCGGTTCTTGTTCTCC-´3, 5´- ACACATCACCTCCTTGTCAGACT-´three and FAM-TGAGCCACCATCACCCAGACACA-BHQ (84). Actin was used as a housekeeping gene and was detected by qPCRBIO SyGreen mix HI-ROX (PCR Biosystems) together with QuantiTect primer assay (QT01680476, Qiagen). Viral RNA was either normalized to actin viral RNA (%) or normalized to actin using the ∆∆Ct method where the fold induction of viral RNA was determined by normalization to the inputs level of viral RNA (at 2 h post infection). All quantitative PCR experiments were performed on the StepOnePlus fast real-time PCR system (Applied Biosystems).

### Proximity ligation assay (PLA)

HEK293T cells and ACBD3 KO cells were seeded and transfected with 1 µg of NS4B-GFP plasmid. After 24 h, the cells were fixed with 4% paraformaldehyde (PFA) for 15 min at RT. To detect interactions between NS4B-GFP and ACBD3, the samples were incubated with primary antibodies against GFP (mouse monoclonal, JL-08, Clontech) and ACBD3 (rabbit polyclonal, Atlas Antibodies) at two dilution combinations: anti-GFP 1:200 with anti-ACBD3 1:500 and anti-GFP 1:100 with anti-ACBD3 1:1000. Technical controls included samples incubated with only one primary antibody or none to monitor nonspecific signal and background fluorescence. PLA was performed according to the manufacturer’s protocol using the Duolink PLA detection kit (FarRed, Sigma-Aldrich). In summary, secondary antibodies conjugated to PLA probes were applied, followed by ligation and rolling circle amplification steps to generate fluorescent puncta indicating protein-protein proximity (< 40 nm). SIM images of PLA signals were acquired using a Zeiss Elyra 7 super-resolution microscope. Quantification of PLA signals was performed using ImageJ software to compare the interaction levels across conditions.

### Malachite green EM sample preparation of TBEV-Infected HEK cells

HEK293T cells and ACBD3 KO cells were seeded onto 35 mm MatTek dishes and cultured overnight to reach ~80% confluency. The cells were then infected with TBEV at a MOI of 1 and incubated for 24 h. Prior to EM sample preparation, the infected dishes were fixed in 4% PFA for 30 min to inactivate the virions. The samples were subsequently crosslinked with a fixative containing 2.5% glutaraldehyde and 0.05% malachite green oxalate in 0.1 M PHEM buffer. Pre-staining was performed using a solution of 0.8% potassium ferricyanide (K₃[Fe (CN)₆]) and 1% osmium tetroxide (OsO₄) in 0.1 M PHEM buffer. This was followed by blocking with 1% tannic acid and staining with 0.5% uranyl acetate. After staining, the samples were washed thoroughly and dehydrated using an ethanol gradient series (25%, 50%, 75%, 90%, 95%, and two steps of 100%). The samples were then infiltrated with a series of resin-to-ethanol mixtures (TAAB resin: EtOH ratios of 1:3, 1:1, and 3:1), followed by a final infiltration step with 100% resin. The resin was replaced with fresh resin, and the samples were polymerized at 60°C overnight. All infiltration and washing steps were performed using a PELCO BioWave Pro+ microwave processor. The processed samples were sectioned, and 75 nm thick sections were acquired. Imaging was conducted using an FEI Talos L120C transmission electron microscope.

### TBEV replicon expression and analysis

HEK293T cells and ACBD3 KO cells were grown on cover glass in 6-well plates and transfected for 24 h with 1 µg of the TBEV-replicon plasmid using Lipofectamine 2000 (Invitrogen) according to the manufacturer’s instructions. Cells on cover glasses were fixed, immunostained as described in immunofluorescence staining. Fluorescent images were acquired using a Leica Thunder microscope with a HC PL APO 63×/1.4 oil CS2 objective (Leica) using the same lamp power and exposure times. Image analysis was performed using ImageJ. The total fluorescent signal from each image was determined (14–20 cells per image), and following the subtraction of background antibody stain, the intensities were divided by the number of transfected cells per image (2–11 cells per image). Transfection efficiency was quantified by manual counting of the total number of cells per image and the number of NS3 positive cells with an intensity over the set intensity threshold. Twelve images per condition (over 600 cells per condition) from three independent experiments were counted, and the mean and SEM and statistical *P*-value (unpaired *t*-test) were calculated using Prism software.

### Virus-like particle secretion assay

HEK293T cells and ACBD3 KO cells were grown in T75 flasks and transfected for 24 h with 3 µg of plasmids encoding for viral proteins C, prM, and E-3xFLAG each (9, 42), or 9 µg of GFP plasmid as control, using GeneJuice transfecting agent (Sigma-Merck) according to the manufacturer’s instructions. The culture was then harvested as cells and supernatants separately. First, the cells were detached using cold PBS and lysed in 350 µL lysis buffer (50 mM Tris pH 8.0, 150 mM NaCl, 1% Triton X-100) containing protease inhibitor (Roche) for at least 20 min on ice. Cell lysate was sonicated for 7 s (Hielscher UP200St) and centrifuged at 20,000 × *g* for 10 min at 4°C. Cleared lysate was transferred to a new tube, mixed with 2× Laemmli buffer (Bio-Rad) containing 100 mM DTT and boiled for 5 min at 95°C for immunoblotting. Second, the culture supernatant harvested was clarified by centrifugation at 500 × *g* for 5 min at 4°C. Pre-cleared supernatant was ultracentrifuged at 100,000 × *g* for 1.5 h at 4°C. The supernatant was then removed. The pellet was resuspended in 50 µL 2 × Laemmli buffer containing 100 mM DTT overnight at 4°C. The samples were boiled for 5 min at 95°C and analyzed with immunoblotting as above. Quantification was performed by normalizing the protein in the VLPs to the protein in the lysate.

### Statistics

For the data of NS4B-APEX2 proteomics, statistical significance was calculated with unpaired *t*-test using Excel (Microsoft). Statistical analyses of the other experiments were performed using Prism 10 (GraphPad). Unpaired *t*-test was used to analyze the data of plate imager-based assays, focus forming assays, RT-qPCR, TBEV replicon, and VLP secretion assay. The Mann-Whitney test was used to analyze the data of confocal images. One-way ANOVA with Dunnett multiple test was used to analyze the data of CRISPR-Cas9 KD assay and rescue assay with ACBD3 mutants. The specific tests used and the *P*-values are indicated in the figure legends.

## Data Availability

The mass spectrometry proteomics data have been deposited to the ProtomeXchange Consortium via PRIDE (85) partner repository with the data set identifier PXD051173.

## SUPPLEMENTAL MATERIAL

Supplemental figures jvi.02224-24-s0001.docx Figures S1 to S4; legends for Table S1 and Movies S1 and S2.

Table S1 jvi.02224-24-s0002.xlsx Mass spectrometry result and gene ontology analysis.

Movie S1 jvi.02224-24-s0003.mp4 3D representations of the volume SIM imaging data of ACBD3 and SEC23IP in uninfected cells.

Movie S2 jvi.02224-24-s0004.mp4 3D representations of the volume SIM imaging data of ACBD3 and SEC23IP in infected cells.

ASM does not own the copyrights to Supplemental Material that may be linked to, or accessed through, an article. The authors have granted ASM a non-exclusive, world-wide license to publish the Supplemental Material files. Please contact the corresponding author directly for reuse.
