# Supplementary material for: The ACBD3 protein coordinates ER-Golgi contacts to enable productive TBEV infection
Source: J Virol. 2025 Apr 10;99(5):e02224-24. doi: 10.1128/jvi.02224-24 (PMC12090792; doi:10.1128/jvi.02224-24)
Supplement: Supplemental figures — Figures S1 to S4; legends for Table S1 and Movies S1 and S2. [file jvi.02224-24-s0001.docx]

**Supporting information**

**The ACBD3 protein coordinates ER-Golgi contacts to enable productive TBEV infection**

Wai-Lok Yau, Marie B. A. Peters, Sebastian Rönfeldt, Marie Sorin, Richard Lindqvist, Lauri I. A. Pulkkinen, Lars-Anders Carlson, Anna K. Överby, Richard Lundmark

**S1 Table. Mass spectrometry result and gene ontology analysis.**

STRING functional enrichment analysis of the NS4B-APEX2 proteomes was performed using STRING version 12.0 (<https://string-db.org/>) [1]. Small geneset-based analysis of protein input with FC value of hits of NS4B-APEX2 infected with LGTV over mock was used and compared with the whole human proteome and at a confidence of interaction score 0.7. Venn diagram of the hits in S1 Table C was performed using BioVenn web application (<https://www.biovenn.nl/index.php>) [2]. ShinyGO enrichment analysis was performed using ShinyGO version 0.77 (<http://bioinformatics.sdstate.edu/go/>) [3] with the parameters p-value of FDR cutoff at 0.05, 10 pathways to show, pathway size from 2 to 100, remove redundancy and abbreviate pathways.


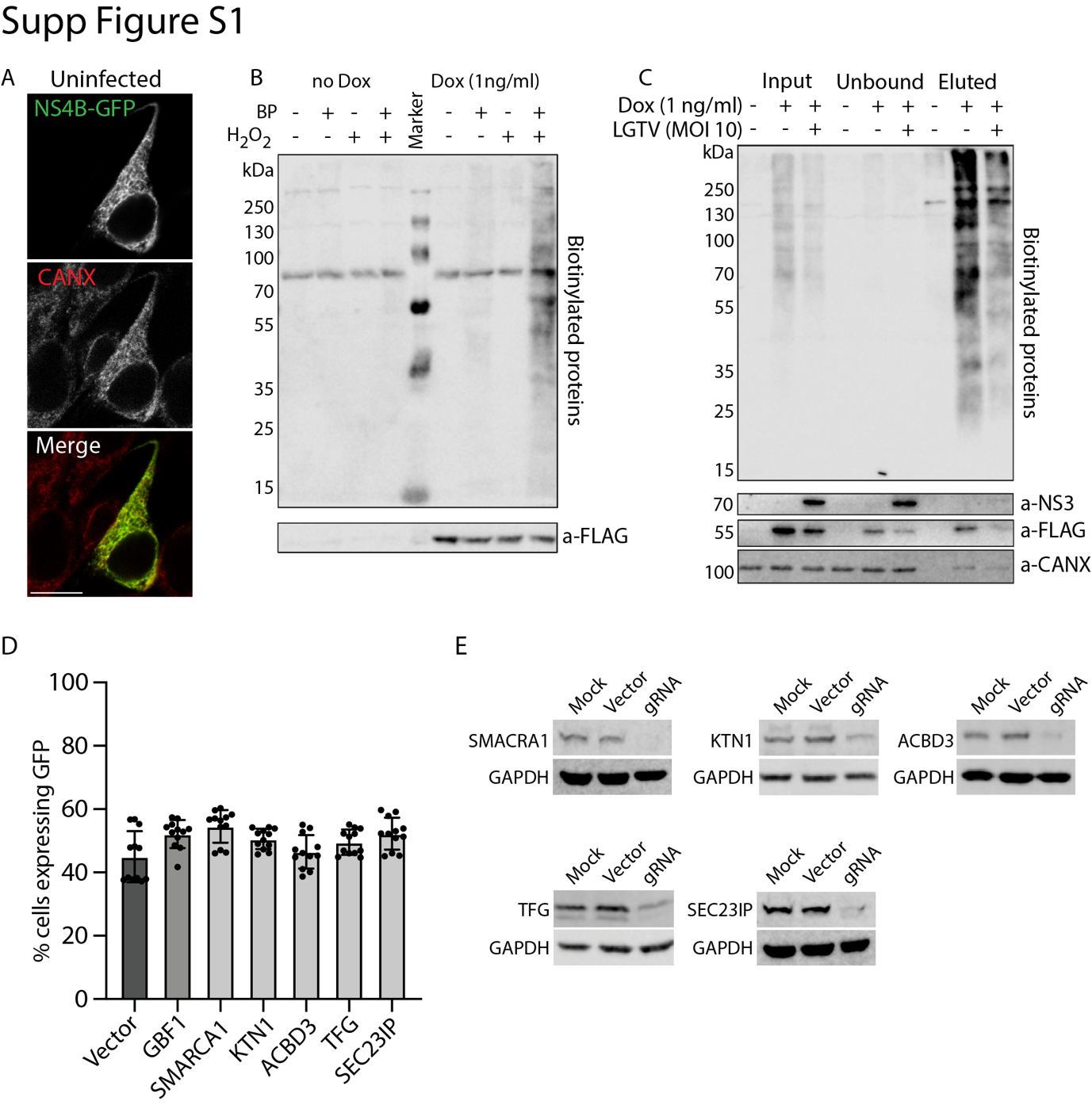


**S1 Fig (related to Fig 1). Validation of NS4B-APEX2 proximal protein analysis.**

(A) Confocal fluorescence micrographs of uninfected HEK293T cells transiently over-expressing NS4B-GFP and stained with anti-CANX antibodies. Scale bar, 10 µm. (B) Immunoblot analysis of NS4B-APEX2 cells treated with Dox and H_2_O_2_ as specified. The cell lysate was probed with streptavidin 680RD for biotinylated proteins and anti-FLAG M2 antibodies for NS4B-3xFLAG-APEX2. (C) Pulldown analysis of LGTV-infected (MOI 10, 16 h.p.i.) and Dox-induced NS4B-APEX2 cells treated for APEX2 biotinylation. The cell lysate was mixed with neutravidin beads to pull down biotinylated proteins. Cell lysate, unbound samples and elute were analyzed by immunoblot with streptavidin 680RD, anti-NS3, anti-CANX, anti-FLAG M2 antibodies. (D) FACS Quantification of the percentage of GFP-expressing HEK293T after CRISPR-Cas9 KD of the indicated proteins. (E) Immunoblot analysis of SMARCA1, KTN1, ACBD3, TFG and SEC23IP KD efficiency. GAPDH was used as loading control.


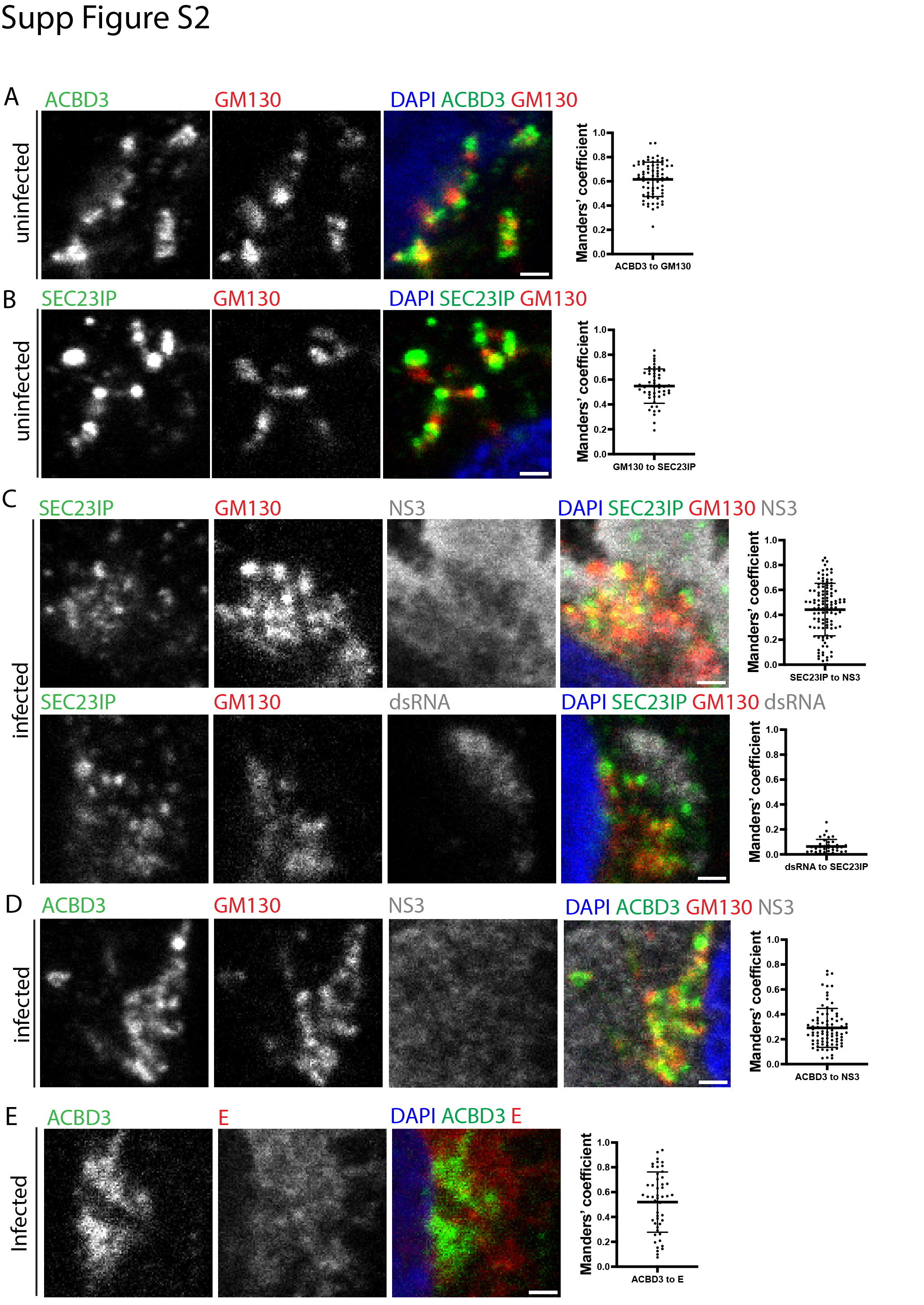


**S2 Fig (related to Fig 2). ACBD3 and SEC23IP localize at the ERES-Golgi contacts.**

(A-B) Confocal fluorescence micrographs of uninfected HEK293T cells stained with anti-ACBD3 antibodies and anti-GM130 antibodies (A) or anti-SEC23IP antibodies and anti-GM130 antibodies. Scale bars, 1 µm. (C-E) Confocal fluorescence micrographs of LGTV-infected (MOI 1, 16 h.p.i.) HEK293T cells stained with antibodies against SEC23IP, GM130, NS3 and dsRNA (C), ACBD3, GM130, NS3 (D), or ACBD3 and E (E) as indicated. Scale bars, 1 µm. Manders’ coefficients between the indicated signals are shown on the right-hand side of each panel as mean ± SD.


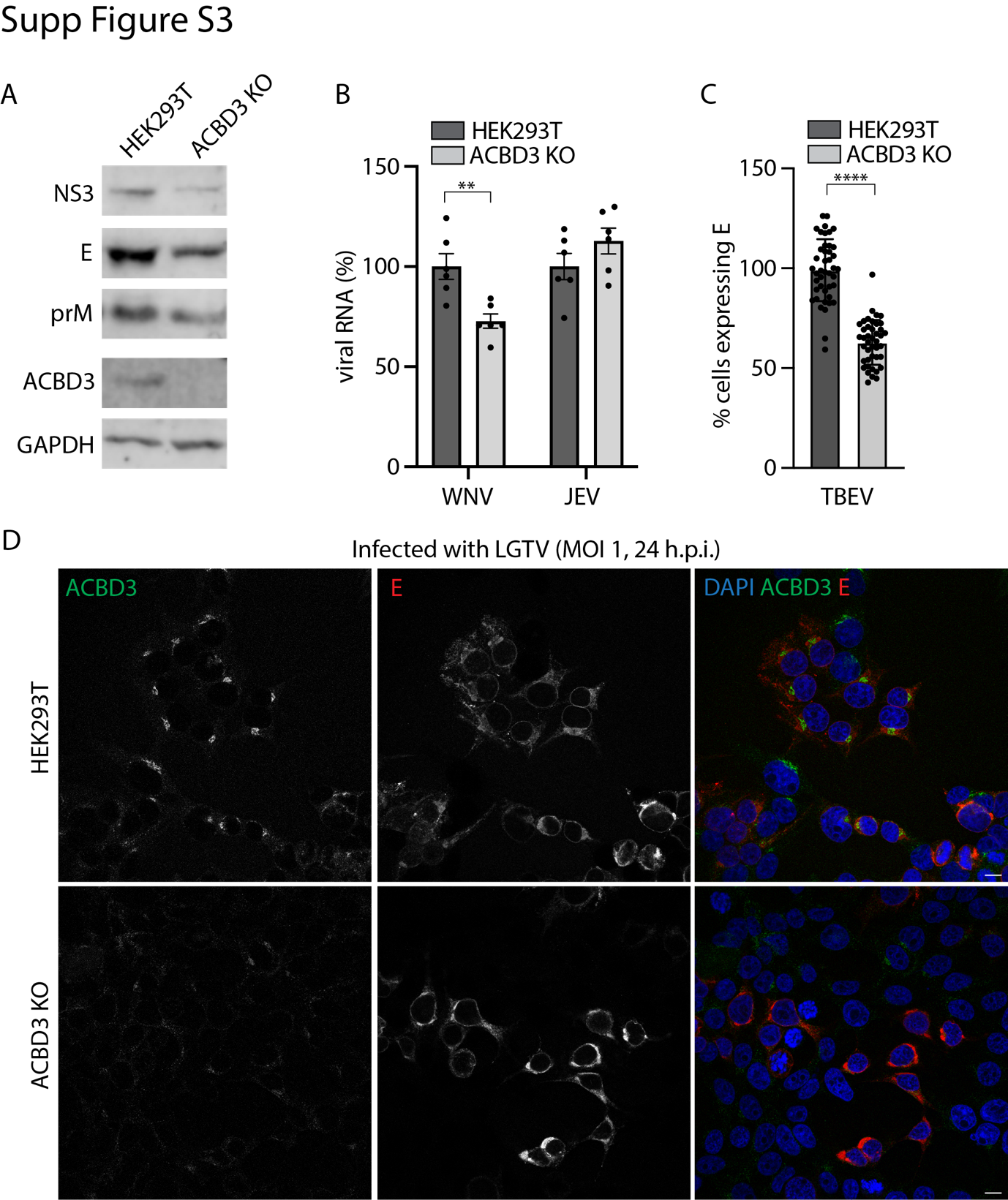


**S3 Fig (related to Fig 3). ACBD3 KO affects tick-borne flavivirus species.**

(A) Immunoblot analysis of NS3, E, prM, and ACBD3 expression in LGTV-infected (MOI 1, 24 h.p.i.) HEK293T and ACBD3 KO cells. GAPDH was used as a loading control. (B) Relative comparison of viral RNA in HEK293T cells and ACBD3 KO cells infected with WNV or JEV at 16 h.p.i., MOI 1. The relative change was calculated as in (Fig 3 C) and the data were normalized to WT HEK293T cells for comparison between viruses. Mean ± SD of 3 biological replicates, unpaired t-test. (C) Quantification of the percentage of WT HEK293T and ACBD3 KO cells expressing E proteins at 24 h after TBEV infection (MOI 1). Mean ± SD of at least 7 biological replicates. Unpaired t-test. * p < 0.05, ** p < 0.005, **** p < 0.0001. (D) Confocal fluorescent micrographs of LGTV-infected (MOI 1) HEK293T and ACBD3 KO cells stained with anti-ACBD3 and anti-E antibodies and DAPI 24h p.i.. Scale bar, 10 µm.


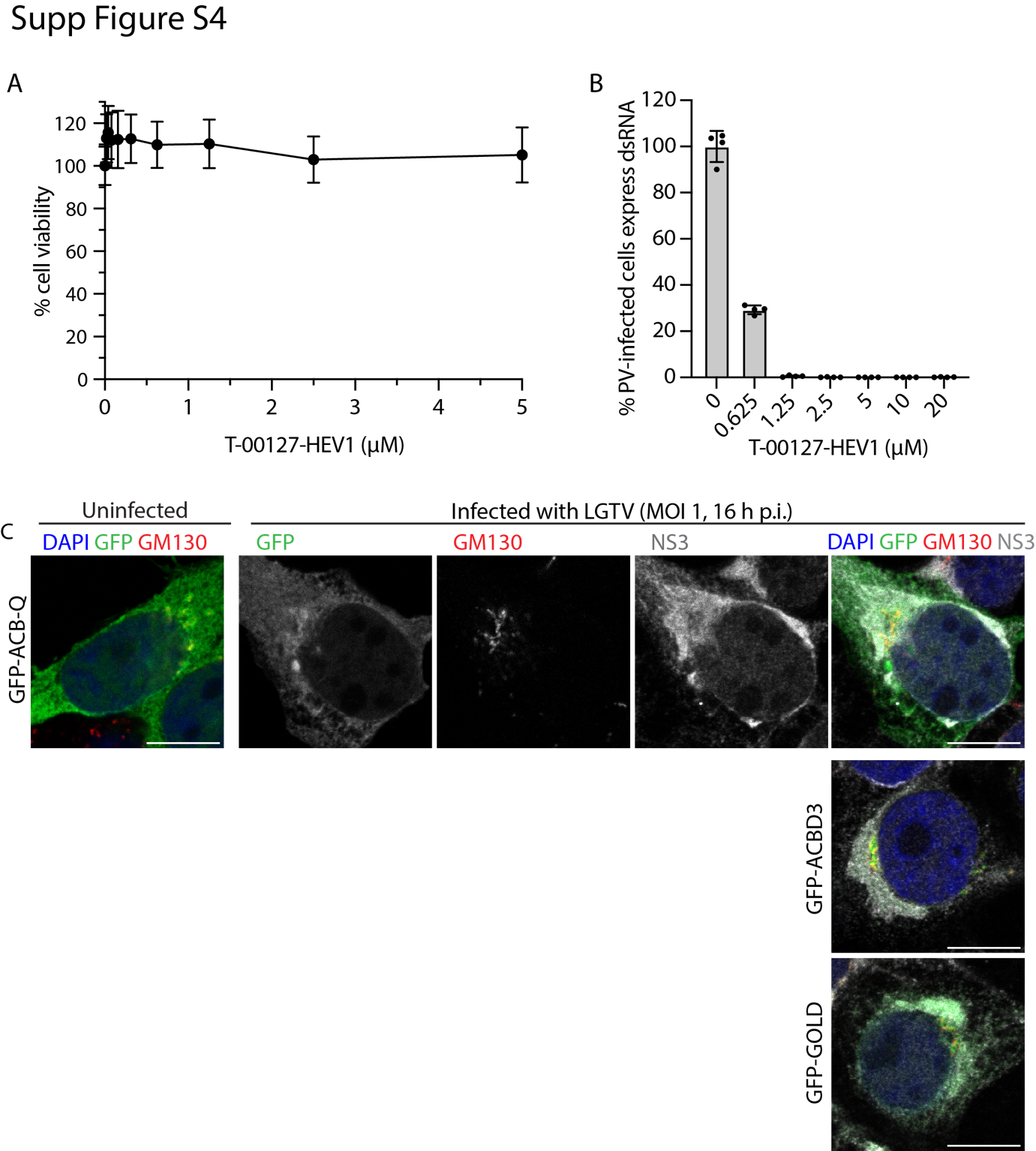


**S4 Fig (related to Fig 6). PI4KB inhibitor T-00127-HEV1 inhibits poliovirus infection.**

(A) Viability test of A549 cells treated with PI4KB inhibitor T-00127-HEV1. A549 cells were treated with T-00127-HEV1 for 24 hours at 37ºC with 5% CO2. Cell viability of the cells was determined with Celltiter Glo luminescent cell viability assay (Promega) and Synergy HT plate reader (Biotek). Mean ± SD of 4 biological replicates from a representative experiment. (B) Quantification of the percentage of A549 cells positive for dsRNA at 6 h.p.i. with PV (MOI 10), in the absence and presence of T-00127-HEV1 (0 – 20 µM). Data were normalized to DMSO-treated A549 control cells. Mean ± SD of 4 biological replicates from a representative experiment. (C) Confocal fluorescence micrographs of ACBD3 KO cells transiently over-expressing GFP-ACB-Q, GFP-ACBD3, or GFP-GOLD stained with anti-GM130 and NS3 (only in the infection experiment) antibodies either without infection (left) or 16 h after LGTV infection at MOI 1 (right). Scale bar, 10 µm.

**S1 movies (related to Fig 3). 3D representations of the volume SIM imaging data of ACBD3 and SEC23IP in uninfected cells.**

HEK293T cells transiently expressing GFP-ACBD3 were stained anti-SEC23IP antibodies. (A) Movie of the Z-stack of the SIM images; (B) Movie of the inset; (C) Movie of the volume SIM imaging data of the inset. Green, GFP-ACBD3; Red, SEC23IP.

**S2 movies (related to Fig 3). 3D representations of the volume SIM imaging data of ACBD3 and SEC23IP in infected cells.**

HEK293T cells transiently expressing GFP-ACBD3 at 16 h.p.i. with LGTV (MOI 1) were stained anti-SEC23IP antibodies and anti-E antibodies. (A) Movie of the Z-stack of the SIM images; (B) Movie of the inset; (C) Movie of the volume SIM imaging data of the inset. Green, GFP-ACBD3; Red, SEC23IP; Gray, E.

**Reference**

1. Szklarczyk D, Kirsch R, Koutrouli M, Nastou K, Mehryary F, Hachilif R, et al. The STRING database in 2023: protein–protein association networks and functional enrichment analyses for any sequenced genome of interest. Nucleic Acids Res. 2022;51: D638–D646. doi:10.1093/nar/gkac1000

2. Hulsen T, Vlieg J de, Alkema W. BioVenn – a web application for the comparison and visualization of biological lists using area-proportional Venn diagrams. BMC Genom. 2008;9: 488. doi:10.1186/1471-2164-9-488

3. Ge SX, Jung D, Yao R. ShinyGO: a graphical gene-set enrichment tool for animals and plants. Bioinformatics. 2020;36: 2628–2629. doi:10.1093/bioinformatics/btz931
